# Adsorption and Separation by Flexible MOFs

**DOI:** 10.1002/adma.202414724

**Published:** 2025-01-28

**Authors:** Irena Senkovska, Volodymyr Bon, Antonia Mosberger, Yutong Wang, Stefan Kaskel

**Affiliations:** ^1^ Chair of Inorganic Chemistry I Technische Universität Dresden Bergstrasse 66 01069 Dresden Germany

**Keywords:** adsorption selectivity, breathing, flexible metal–organic frameworks, gate opening, gas separation, gas storage, soft porous crystals

## Abstract

Flexible metal–organic frameworks (MOFs) offer unique opportunities due to their dynamic structural adaptability. This review explores the impact of flexibility on gas adsorption, highlighting key concepts for gas storage and separation. Specific examples demonstrate the principal effectiveness of flexible frameworks in enhancing gas uptake and working capacity. Additionally, mixed gas adsorption and separation of mixtures are reviewed, showcasing their potential in selective gas separation. The review also discusses the critical role of the single gas isotherms analysis and adsorption conditions in designing separation experiments. Advanced combined characterization techniques are crucial for understanding the behavior of flexible MOFs, including monitoring of phase transitions, framework–guest and guest–guest interactions. Key challenges in the practical application of flexible adsorbents are addressed, such as the kinetics of switching, volume change, and potential crystal damage during phase transitions. Furthermore, the effects of additives and shaping on flexibility and the “slipping off effect” are discussed. Finally, the benefits of phase transitions beyond improved working capacity and selectivity are outlined, with a particular focus on the advantages of intrinsic thermal management. This review highlights the potential and challenges of using flexible MOFs in gas storage and separation technologies, offering insights for future research and application.

## Graphical abstract

Flexible metal–organic frameworks (MOFs) present significant potential for gas storage and separation due to their structural dynamic. This review explores the rationale behind the flexible MOFs' enhanced working capacity and separation factors. It also addresses key challenges, including phase transition kinetics, crystal robustness, cycling, shaping, and thermal management, and highlights advanced characterization methods essential for global understanding of flexibility phenomena.

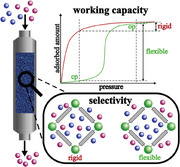

## 1 Introduction

Metal–organic frameworks (MOFs) are crystalline coordination polymers composed of metal ions or clusters coordinated by organic ligands, facilitating the formation of inner cavities.^[^ ^1^ ^]^ Due to their dual inorganic/organic nature as coordination compounds, the chemistry of MOFs is enormously rich. The vast number of metal/ligand combinations grants these compounds exceptional versatility, unlocking immense potential for a wide range of applications, including storage and separation of molecular species.^[^ ^2^ ^]^ However, the vast diversity also turns MOFs into a “haystack,” making it challenging for researchers to find the “needle” when trying to identify the best MOF for a specific application.

Recognized early on for their potential as porous materials, MOFs have undergone significant advancements since their inception. At the early stage of development, MOFs faced challenges with maintaining permanent porosity, and initial structures often collapsed after removing guest molecules. However, advances in desolvation techniques^[^ ^3^ ^]^ and a better understanding of building principles^[^ ^4^ ^]^ led to the development of robust porous MOFs, among others, with enhanced chemical stability. After this breakthrough, MOFs became prominent for their ability to adsorb and separate gases.

The primary interest in MOFs is, nonetheless, motivated by their modular construction and accessibility to deliberate structural design and reticular chemistry.^[^ ^5^ ^]^ Access to isoreticular families of materials allowed for systematic studies of structure–properties relationships.^[^ ^6^ ^]^ The result of these designs is ultrahigh porosity with surface areas of up to 7850 m^2^ g^−1^ and pore volume of 5.0 cm^3^ g^−1^.^[^ ^7^ ^]^ MOFs still hold records for specific surface area and pore volume among microporous adsorbents. Such new horizons in terms of porosity and storage capacities catapulted MOFs to high‐performance adsorbents.^[^ ^8^ ^]^

However, the main feature distinguishing MOFs from other porous adsorbents, such as activated carbons, is not only the crystallinity of these materials and, as a consequence, the regular and crystallographically precise pore structure. A unique feature of some MOFs is the structural flexibility and the ability to undergo stimuli‐induced structural transitions.^[^ ^9^ ^]^ Some MOF representatives are able to switch between the crystalline phases with different porosity induced by guest physisorption. A number of terms have been proposed to manifest the flexibility of MOFs, including “soft,” “flexible,” “dynamic,” “stimuli‐responsive,” or “switchable.” They all describe solid‐state structural phase transitions initiated by relatively small energetic stimuli and usually (but not necessarily) include considerable changes in the unit cell and pore volumes.^[^ ^9^, ^10^ ^]^

They are also emerging material classes, including covalent organic frameworks (COFs)^[^ ^11^ ^]^ and hydrogen‐bonded organic frameworks (HOFs),^[^ ^12^ ^]^ sharing with MOFs key characteristics, such as crystallinity, porosity, and ability to undergo structural transitions. While this review focuses on MOFs, the general observations and dependencies discussed here are likely applicable to these materials as well.

Although the variety of phase transitions inducing stimuli is broad, including pressure, temperature, electric field, light etc.,^[^ ^13^ ^]^ we are going to focus on the structural changes caused by the adsorption or desorption of molecules and the benefits/drawbacks of them in fields of gas storage and separation.

**Figure 1:**
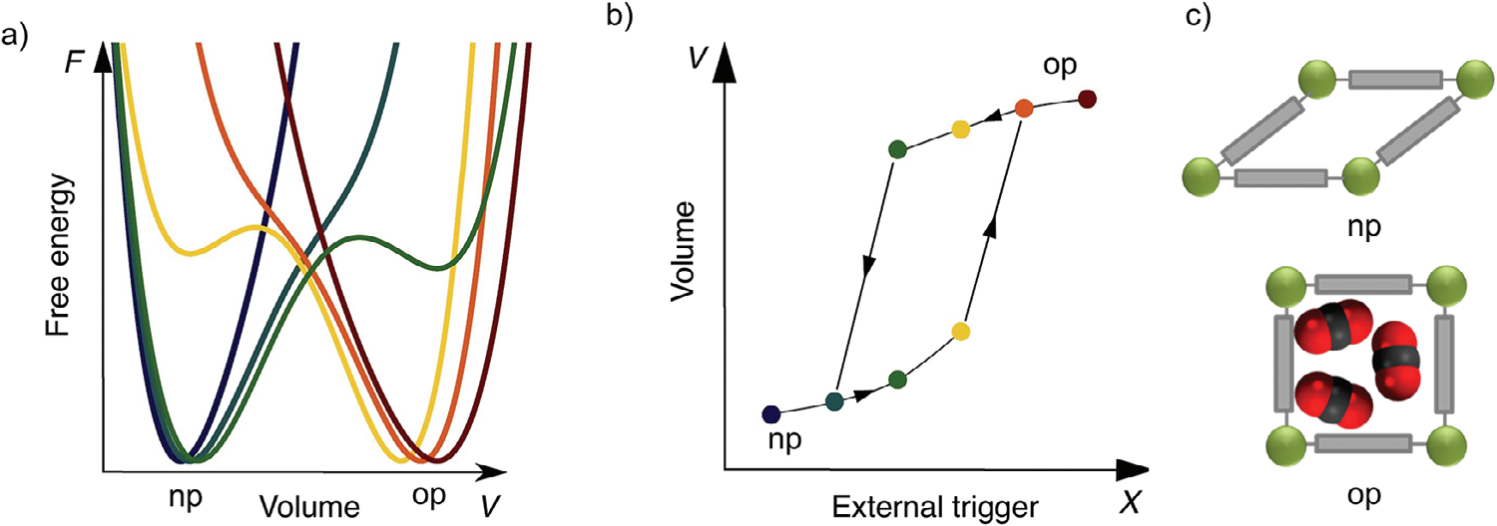
Schematic representations of the structural transitions between narrow pore phase (np) and open pore phase (op) induced by a general state variable *X*: a) free energy *F*(*V*; *X*); b) response V(*X*), Reproduced with permission.^[^ ^16^ ^]^ Copyright 2019, Elsevier Ltd.; as well as schematic representation of the two corresponding framework states (c).

In general, the structural transitions lead to a stepwise increase (or decrease) of porosity between two extreme cases:^[^ ^14^ ^]^ i) nonporous state (closed pore phase, cp), resulting from the dense packing of the building blocks and ii) maximal possible pore size and volume for given framework topology (open pore phase, op). The number of (meta)stable crystal structures (intermediate phases (ip), or narrow pore phases (np)) between these two extremes in the given system depends on the free energy landscape for the given fluid/framework combination, e.g., the number of local minima and the height of activation barriers that separate them (multistability of free energy profile, **Figure** **1**a). The existence of the energy barrier between two minima in the free energy landscape is the origin of hysteresis loops in the adsorption/desorption isotherms (Figure 1b).^[^ ^15^ ^]^

Depending on the initial state of the framework and the transition pathway, the “gate‐opening” and “breathing” transitions are mainly discussed. Sometimes, the authors also use the terms interchangeably. “Gate‐opening” behavior is characterized by the increase in the porosity upon the transition, whereby a less porous structure (cp or np) expands to a more porous after a certain threshold gas pressure (*p*_go_) is reached. The physisorption isotherm, in this case, is characterized by the stepwise increase in the adsorbed amount in a pretty narrow pressure region (Figure 1c).

“Breathing” usually includes two consecutive transitions during gas pressure increase: first contraction transition of the more porous phase (op) to the less porous one (np) at characteristic contraction pressure (*p*_c_), followed by second expansion transition (gate opening) to a more porous (large pore or op) phase at characteristic *p*_ex_. The first contraction is usually not easily identified in the isotherm (particularly if the phases have a minor porosity difference) and have to be often detected by complementary techniques, such as in situ powder X‐ray diffraction (PXRD) (see Section 3).

The isotherms of flexible MOFs can be constructed by superimposing the isotherms of two or more structures with varying adsorption capacities, adsorption enthalpies, etc. However, typically along the isotherm, the metastable states are observed. The adsorption or desorption branch (or both) can be kinetically hindered, and the energetic balance strictly depends on the number of guest molecules in the pores. Thus, the global minimum state, among all possible configurations, changes depending on the external guest pressure.^[^ ^17^ ^]^ These activation barriers stem 1) from the solid–solid phase transitions and are particularly high if the volume change is high, but also 2) the fluid‐phase activation barriers (diffusion, nucleation, etc.) may play a role. These factors lead to hysteresis, which does not vanish even for long equilibration times because the barriers depend exponentially on one or more thermodynamic variables and can only be overcome if the variable reaches a critical magnitude (such as gate opening pressure, see below). Solid–solid phase transitions with high activation barriers may be characterized as 1^st^ order transitions leading to steps in the isotherm. If the barriers for solid–solid transitions are minimal, a quasi‐continuous structural change is observed, particularly when the volume changes are minor because the pores are already filled with linker functionalities.^[^ ^18^ ^]^

The fascination of flexible MOFs for separations originates from the vision that an MOF may recognize a molecular stimulus so specifically that it opens the pores only for this species, even if it is only a minor component in a mixture of molecules, similar to an enzyme recognizing its substrate.^[^ ^19^ ^]^ However, the benefits of the dynamic nature of MOFs for adsorptive application are controversially discussed in the literature. Besides the benefits of structural flexibility discussed below in detail (Sections 2.1–2.3), there are some critical material‐relevant, as well as technological aspects related to flexibility. The primary concerns involve the cyclability and the damage of the crystals in the course of multiple expansion/shrinkage cycles (Section 4.3) and the management of the crystal volume change in the adsorption chamber or column (Section 4.2). Since the first discovery of switchable MOFs, the number of new MOFs showing pronounced flexibility and huge changes in pore size is increasing exponentially. Also, some unique phenomena connected to gas‐induced flexibility, such as “negative gas adsorption” were discovered, and, despite open questions, more and more functions resulting from pore switchability are reported. In the following, we discuss the concepts of using flexible MOFs as efficient adsorbents, giving some examples.

## 2 Impact of Flexibility and Concepts for Gas Adsorption and Separation Using Flexible MOFs

### 2.1 Gas Storage Concept

Gas storage is one of the most essential applications of porous materials, based on gas enrichment in an interfacial layer due to solid–fluid interactions.

Volumetric and gravimetric capacities are of central importance for gas storage, although the temperature of operation and thermal effects upon adsorption are also crucial. The aim is, therefore, to achieve high volumetric and gravimetric capacities at a usable temperature.^[^ ^20^ ^]^

The main indicator for the comparison of the storage performance, however, is the so‐called working (also usable) capacity, reflecting the difference between the amount of gas adsorbed at the target storage pressure and the amount that still be adsorbed at the lowest desorption pressure, acceptable for the operation of the system. In rigid adsorbents, the best performance can be reached by the adjustment of the pore sizes to the operational pressure and temperature range. The subatmospheric pressures require (ultra)microporosity, whereas the mesoporous MOFs or MOFs with hierarchical pore structure are beneficial for high‐pressure storage.^[^ ^21^ ^]^

**Figure 2:**
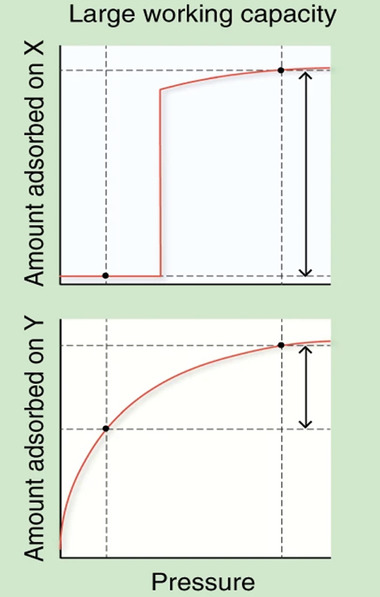
Comparison of the typical adsorption isotherms of flexible MOF (*X*, top) and rigid MOF with comparable pore size (*Y*, bottom). The working capacity in both cases is indicated by the arrow. Reproduced with permission.^[^ ^22^ ^]^ Copyright 2020, The Authors, under CC BY 4.0.

The stepwise isotherms, characteristic for flexible MOFs, open a new opportunity for boosting usable capacity, where the amount of gas adsorbed would be negligible at low pressures (in the pressure region below the lowest desorption pressure acceptable) but rise sharply just before the pressure reaches the desired storage pressure (**Figure** **2**). Flexibility allows the material to close pores at relatively high pressure and expel the gas molecules from the pores. As a consequence, more gas can be released from the pores in comparison to the rigid MOF with a comparable pore size. In the pressure region above that is needed for the opening transition, the gas uptake is equal to that of comparable microporous adsorbent. Therefore, the optimal opening and closing pressures are crucial for achieving high usable capacity. A critical aspect is hysteresis, which should be as narrow as possible to achieve a high working capacity and reduce energy consumption.

The second benefit offered by flexible MOFs is thermal management. There are practical challenges involved in designing systems with high capacities and managing the temperature change associated with the adsorption and desorption of gas from the adsorbent due to the high gas adsorption enthalpies in microporous materials. In practical gas storage application systems, the heat of adsorption (exothermic) and desorption (endothermic) can lead to strong temperature variations,^[^ ^23^ ^]^ which can have a negative impact on the usable capacity. Rapid charging and discharging are desirable, but they also lead to significant temperature fluctuations. For example, in the case of CH_4_ storage, measurements with commercially available Adsorbed Natural Gas (ANG) cylinders show a temperature drop of up to 37 °C at high discharge rates, with performance loss approaching 25% of the isothermal capacity. The performance loss is expected to be 15–20% at moderate discharge rates.^[^ ^24^ ^]^ Analysis indicates that the thermal capacity of the vessel and external heat transfer conditions significantly affect system behavior. MOFs have very low thermal conductivity,^[^ ^25^ ^]^ so additional efforts are required to address thermal management challenges.

In flexible MOFs, fortunately, the enthalpy associated with the phase transition (absorption or release of heat upon reversible phase transitions) can counteract the thermal effect of adsorption, contributing to positive thermal management (Section 5).^[^ ^26^ ^]^ Net heat removed from the flexible system during the desorption process under adiabatic conditions is the sum of the endothermic heat associated with guest desorption, the exothermic heat generated by host shrinkage, and the exothermic heat of the guest molecules that remain in the host framework due to changes in host–guest interactions upon shrinkage of the host.^[^ ^17^ ^]^

Last but not least, flexible MOFs could potentially minimize the “ageing” of adsorbent generated by the accumulation of impurities. The selectivity in adsorption (discussed in detail in Section 2.2) could lead to the adsorption of only the main, valuable components from the gas mixture.

For example, the adsorptive storage of methane often suffers from the adsorption of impurities present in natural gas, such as higher hydrocarbons (ethane, propane, etc.). These hydrocarbons can have a deleterious effect due to accumulation, decreasing methane storage capacity, and affecting the long‐term stability of the adsorbent.^[^ ^27^ ^]^ The selective adsorption of the main component or forced desorption of the impurities supported by the flexibility could improve long‐term performance.

#### 2.1.1 Examples of Flexible MOFs in Storage Applications

In this section, we explore the unique and critical features of flexible MOFs in terms of usable capacity for gas storage and highlight the advantages of flexible MOFs through specific examples. Many gases have been used to study the adsorption behavior of flexible porous materials, but most are not considered for storage applications. This is partly because of well‐developed storage technologies, e.g., compressed Ar, O_2_, N_2_. The gas storage in porous solids is mainly motivated by the goal of increasing the amount of gas within a given volume, e.g., for applications in the mobility sector. Consequently, only a limited number of gases, notably H₂ and CH₄, are typically considered for practical storage purposes.

An additional motivation is the safe storage of hazardous gases, such as acetylene.

##### CH_4_ Storage in Flexible MOFs

As the primary constituent of natural gas, CH_4_ offers a high calorific value, low carbon dioxide emissions, and a high research octane number, making it an attractive fuel for vehicular applications. The Advanced Research Projects Agency‐Energy (ARPA‐E) under the U.S. Department of Energy (DOE) has established several ambitious targets for on‐board CH_4_ storage systems.^[^ ^21^, ^28^ ^]^ The gravimetric storage target is set at 0.5 g(CH₄) per 1 g of adsorbent. The volumetric target (deliverable capacity) is 263 cm^3^ (at standard temperature and pressure, STP) per mL of adsorption chamber at 298 K and 65 bar, corresponding to compressed methane gas at 250 bar.^[^ ^28^, ^29^ ^]^ Additionally, accounting for up to 50% volume loss due to the low packing densities of MOFs,^[^ ^21^ ^]^ the volumetric storage target for the material itself should be even higher. Over the past three decades, MOFs have been extensively investigated and documented for their substantial potential in CH_4_ storage applications.^[^ ^21^, ^30^ ^]^ Computational calculations demonstrated, however, that the usable (deliverable) capacity of almost all conventional rigid adsorbents would not be higher than ≈200 cm^3^ methane per cm^3^ of adsorbent (defined as STP volume at 298 K and 65–5.8 bar pressure window),^[^ ^21^, ^31^ ^]^ which is very low compared to the DOI target. One of the reasons is that most adsorbents exhibit Type I isotherms, where a substantial amount of methane remains adsorbed in the microporous frameworks at pressures around 5 ‐ 6 bar.

Recently, flexible MOFs have gained prominence due to their ability to markedly increase the usable storage capacity. Methane storage in flexible MOFs has been recently summarized by Forrest et al.,^[^ ^32^ ^]^ therefore we will discuss only a few examples in the following.

**Figure 3:**
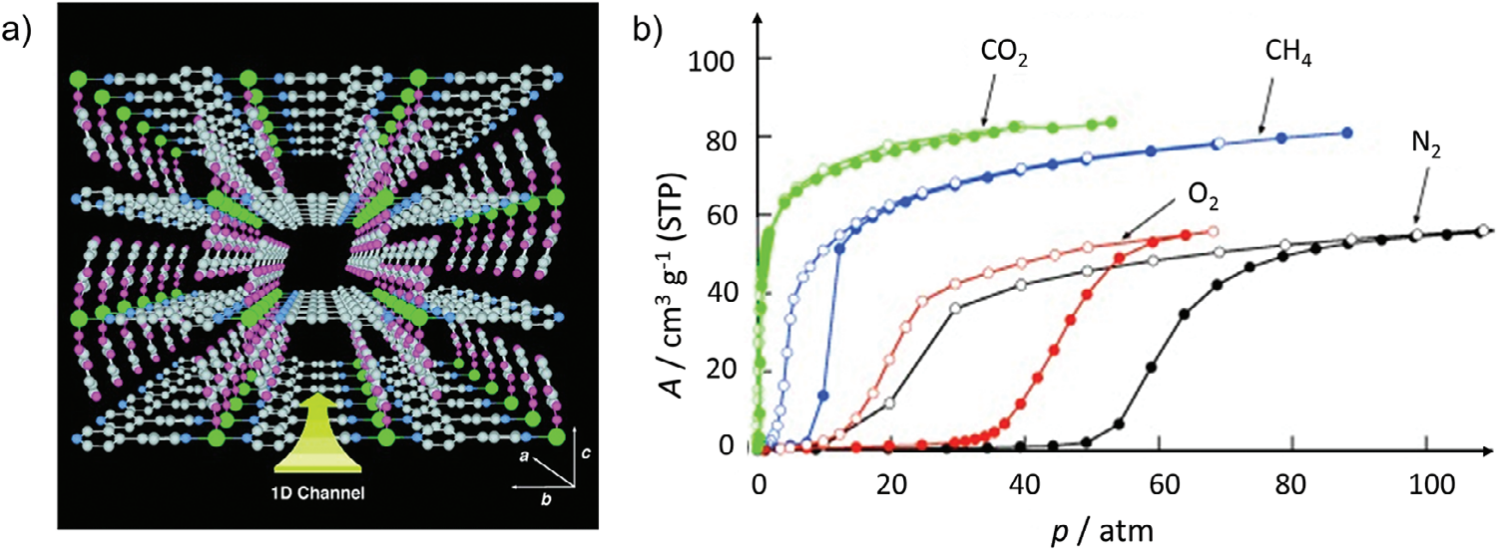
a) Pillared layer structure and b) adsorption (filled circles) and desorption (open circles) isotherms of N_2_, CH_4_, CO_2_ and O_2_ at 298 K for [Cu(dhbc)(4,4′‐bipyridine)]*_n_*. Reproduced with permission.^[^ ^33^ ^]^ Copyright 2002, Wiley‐VCH GmbH & Co. KGaA.

In 2002 and 2003, Seki and Kitagawa reported for the first time a flexible pillared layer MOFs, [Cu_2_(bdc)_2_(4,4′‐bipyridine)]*_n_* (bdc = terephthalate) and [Cu(dhbc)(4,4′‐bipyridine)]*_n_* (Hdhbc  =   2,5‐dihydroxybenzoate), showing the gate opening CH_4_ adsorption isotherm at high pressures and 298 K (**Figure** **3**).^[^ ^33^ ^]^ Although the overall methane uptake is not high, the gate opening and closing pressures are below 10 bar; thus, this reports opened up new avenues for methane storage in flexible frameworks.^[^ ^32^ ^]^

In 2009, Kaneko and co‐workers^[^ ^34^ ^]^ reported methane adsorption isotherm of ELM‐11 ([Cu(BF_4_)_2_(4,4′‐bipyridine)_2_]*_n_*) (Figure 37b,g), and discussed the benefits of gating isotherm for efficient methane storage. The adsorption capacity of the MOF at 65 bar is 55 mg g^−1^. Taking the packing density of the material into account, the storage capacity was calculated to be 155 cm^3^ cm^−3^ (volume of gas/volume of storage vessel).

**Figure 4:**
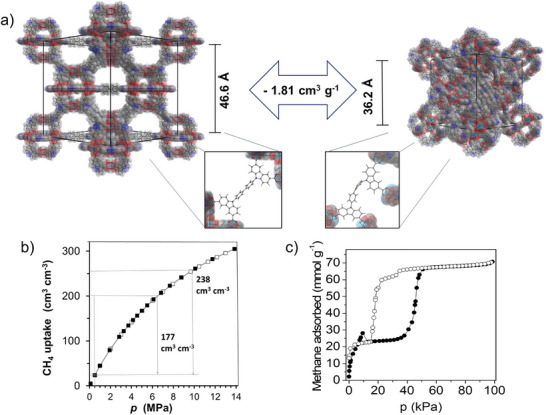
a) Structure and conformation of the DUT‐49 framework and 9,9′‐([1,1′‐biphenyl]−4,4′‐diyl)bis(9H‐carbazole‐3,6‐dicarboxylate) linker in op (left) and cp (right) structures. Guest molecules are omitted for clarity. Reproduced with permission.^[^ ^35^ ^]^ Copyright 2016, Nature Publishing Group. b) Total volumetric CH_4_ adsorption isotherm of DUT‐49 at 298 K (adsorption = closed symbols, desorption = open symbols). The working capacity is given between 65 and 5 bar, and 100 and 5 bar. c) CH_4_ adsorption and desorption isotherms in DUT‐49 at 111 K. Reproduced with permission.^[^ ^35^ ^]^ Copyright 2016, Springer Nature Limited.

In 2012, Stoeck et al.^[^ ^21b^ ^]^ synthesized a carbazole‐based mesoporous MOF (DUT‐49) exhibiting a hierarchical pore system and showing an exceptionally high specific surface area of 5476 m^3^ g^−1^ and a large total pore volume of 2.91 cm^3^ g^−1^, as well as record gravimetric total methane uptake of 0.56 g g^−1^ (236 cm^3^ cm^−3^) at 298 K and 110 bar (**Figure** **4**b). The working capacity of the MOF in the 5–65 bar range amounts to 177 cm^3^ cm^−3^.

Later, authors recognized that DUT‐49 displays an unusual breathing type isotherm with a negative step in the adsorption branch, coined as “negative gas adsorption” (NGA) in methane isotherm measured at 111 K (Figure 4c).^[^ ^35^ ^]^ NGA is recognized as a new, counterintuitive phenomenon based on the release of already adsorbed gas upon contraction of metastable open pore phase of DUT‐49 to a narrow pore phase, accompanied by the reduction of the pore volume of the structure by 1.8 cm^3^ g^−1^.

Krause et al.^[^ ^36^ ^]^ discovered that by varying the length of the linker, the number of CH_4_ molecules adsorbed at the intersection of the open‐pore and closed‐pore phases increased with the ligand length. This leads to a more favorable overall change in adsorption enthalpy for structural contraction, thereby promoting the occurrence of NGA. Then, they investigated the effects of temperature and adsorbate on the occurrence and extent of NGA in DUT‐49.^[^ ^37a^ ^]^ Through experiments covering a broad range of gases and temperatures, the specific temperature ranges in which NGA is observable for each guest molecule could be determined. These findings were further complemented by molecular simulations to explain the absence of NGA at higher temperatures and the non‐monotonic behavior observed at lower temperatures. Although the effect disappears in the methane adsorption isotherm at room temperature, in future, it can be explored for the separation of other gases, such as C3–C4 hydrocarbons, which provoke NGA transitions at room temperature.

**Figure 5:**
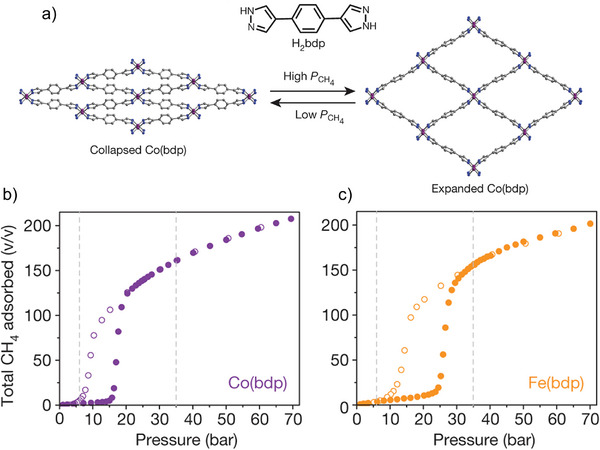
a) The structure of H_2_bdp ligand along with the crystal structures of the closed pore (left) and open pore (right) [Co(bdp)]*_n_* phases. b,c) Total CH_4_ adsorption isotherms for [Co(bdp)]*_n_* (b) and [Fe(bdp)]*_n_* (c) at 298 K. Filled circles represent adsorption; open circles represent desorption. Reproduced with permission.^[^ ^26^ ^]^ Copyright 2015, Nature Publishing Group.

In 2015, Long and co‐workers reported the [*M*(bdp)]*_n_* (bdp = 1,4‐benzenedipyrazolate, **Figure** **5**a) series of MOFs (*M* = Co, Fe) displaying working capacities for CH_4_, very close to the highest working capacities among all MOF materials reported up to now,^[^ ^32^ ^]^ owing to their large pore volume and a high degree of flexibility.^[^ ^26^ ^]^ The [Co(bdp)]*_n_* shows negligible CH_4_ uptake at low pressures, with a noticeable step in the adsorption isotherm after reaching 16 bar (Figure 5b). Desorption exhibits hysteresis, and the loop closes at 7 bar, indicating a reversible structural phase transition (Figure 5a), stable over 100 adsorption/desorption cycles. The CH_4_ usable volumetric storage capacity of [Co(bdp)]*_n_* is 197 cm^3^ cm^−3^ for 5–65 bar pressure range at 25 °C, calculated using the crystallographic density of the materials and geometric pore volume of the expanded phase. High working capacity is explained by nearly complete desorption of the methane from the pores (remind adsorbed amount less than 0.2 mmol g^−1^ at 25 °C) below gate closing pressure.

For isoreticular [Fe(bdp)]*_n_*, opening and closing transitions occur at 24 and 10 bar, respectively (Figure 5c). Nonetheless, [Fe(bdp)]*_n_* maintains a high volumetric usable capacity of 190 cm^3^ cm^−3^ at 5–65 bar and 298 K. Additionally, [Fe(bdp)]*_n_* undergoes further expansion at pressures above 40 bar, forming an op framework with nearly perfect square channels.^[^ ^26^ ^]^

**Figure 6:**
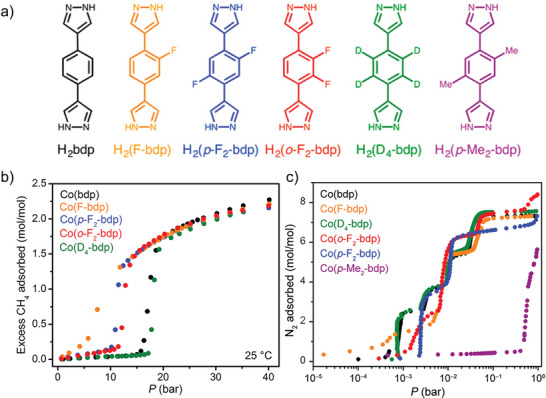
a) The H_2_bdp derivatives used as linker. b) CH_4_ adsorption isotherms of [Co(bdp)]*_n_*, [Co(Fbdp)]*_n_*, [Co(p‐F_2_‐bdp)]*_n_*, [Co(o‐F_2_‐bdp)]*_n_*, and [Co(D_4_‐bdp)]*_n_* at 298 K. c) N_2_ adsorption for [Co(bdp)]*_n_* and derivatives at 77 K. Reproduced with permission.^[^ ^38^ ^]^ Copyright 2016, American Chemical Society.

The same research group proposed a strategy to adjust the pressure required for structural transformation by chemical modification of the linker, which led to five functionalized compounds (**Figure** **6**a), isostructural to [Co(bdp)]*_n_*, namely, [Co(F‐bdp)]*_n_*, [Co(p‐F_2_‐bdp)]*_n_*, [Co(o‐F_2_‐bdp)]*_n_*, [Co(D_4_‐bdp)]*_n_*, and [Co(p‐Me_2_‐bdp)]*_n_*.^[^ ^38^ ^]^ Experimental results indicate that the functionalization of the ligand by introducing fluorine atoms in the phenyl ring leads to a decrease in phase transition pressure by disrupting edge‐to‐face π–π interactions, while methyl groups increase the opening pressure by enhancing these interactions (Figure 6b,c).

**Figure 7:**
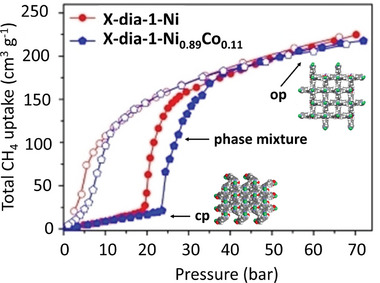
High‐pressure CH_4_ physisorption isotherms for X‐dia‐1‐Ni and X‐dia‐1‐Ni_0.89_Co_0.11_ at 298 K. Adapted with permission.^[^ ^40^ ^]^ Copyright 2023, Wiley‐VCH GmbH.

Yang et al.^[^ ^39^ ^]^ reported a [NiL_2_]*_n_* (L = 4‐(4‐pyridyl)‐biphenyl‐4‐carboxylate), flexible MOF with a **dia** topology and sixfold interpenetration, denoted as X‐dia‐1‐Ni. The framework demonstrates substantial flexibility, and the initially nonporous X‐dia‐1‐Ni underwent multiple phase transitions during CO_2_ adsorption at 195 K (for more crystallographic details, see Section 3.1). High‐pressure CH_4_ adsorption studies at 298 K revealed an S‐shaped adsorption isotherm (**Figure** **7**), with negligible CH_4_ uptake below 20 bar, followed by a sharp increase as the pores open. At 298 K, the working CH_4_ capacity is 162 cm^3^ g^−1^ (147 cm^3^ cm^−3^) at 1 – 65 bar.^[^ ^39^, ^40^ ^]^

However, low closing pressure observed in the desorption isotherms significantly reduces the working capacities, considering the working pressure window between 5 and 65 bar. Cobalt doping in this system (similar to DUT‐8(Ni)^[^ ^41^ ^]^) enables control over the gate opening. So, the methane‐induced phase transformations can be fine‐tuned by using different Ni/Co ratios to enhance methane working capacity.^[^ ^40^ ^]^ The hysteresis was shifted to a higher pressure, and the amount of CH_4_ retained at 5 bar during desorption decreased from 60 cm^3^ g^−1^ in X‐dia‐1‐Ni to 19 cm^3^ g^−1^ in X‐dia‐1‐Ni_0.89_Co_0.11_. Therefore, the working capacity of X‐dia‐1‐Ni_0.89_Co_0.11_ reached 202 cm^3^ g^−1^ (5–65 bar) at 298 K (Figure 7).

**Figure 8:**
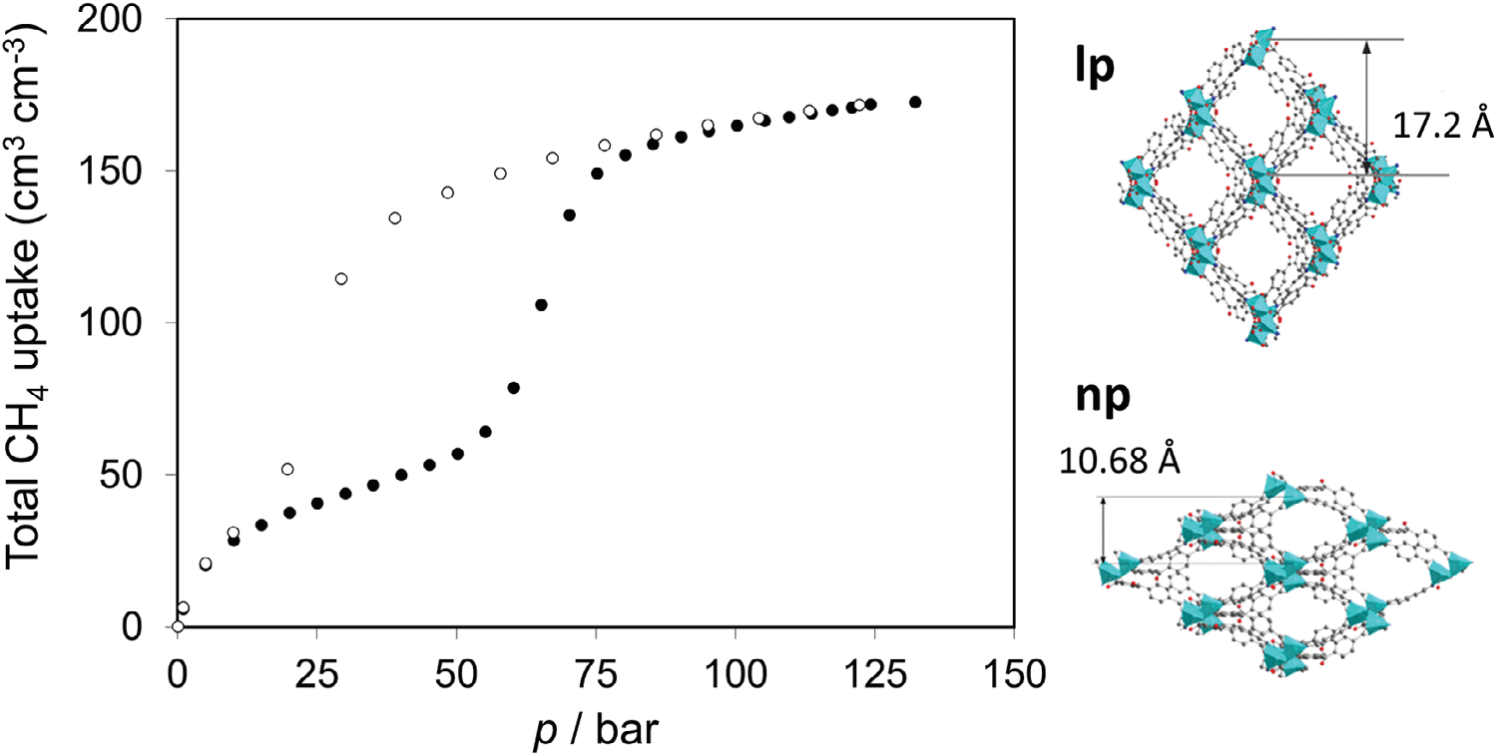
Total methane physisorption isotherm for SNU‐9 at 298 K. Reproduced with permission.^[^ ^44^, ^45^ ^]^ Copyright 2019, Springer Nature Singapore Pte Ltd, Copyright 2014, American Chemical Society.

Kaskel and co‐workers investigated high‐pressure CH_4_ adsorption at 298 K of [Zn_2_(BPnDC)_2_(bpy)]*_n_* (SNU‐9, BPnDC = benzophenone 4,4′‐dicarboxylate, bpy = 4,4′‐bipyridine), the framework reported by Park and Suh already in 2010.^[^ ^42^ ^]^ The compound demonstrates stepwise adsorption isotherm and hysteresis between the adsorption and desorption branches.^[^ ^43^ ^]^ Although the pore volume of this interpenetrated framework is moderate (0.37 cm^3^ g^−1^), the working capacity between 100 and 5 bar at 298 K is 144 cm^3^ cm^−3^ due to the relatively high crystallographic density of the material (1.074 cm^3^ g^−1^ in the open pore state). In the window between 65 and 5 bar it is, however, much lower because of the high gate opening pressure (**Figure** **8**).

**Figure 9:**
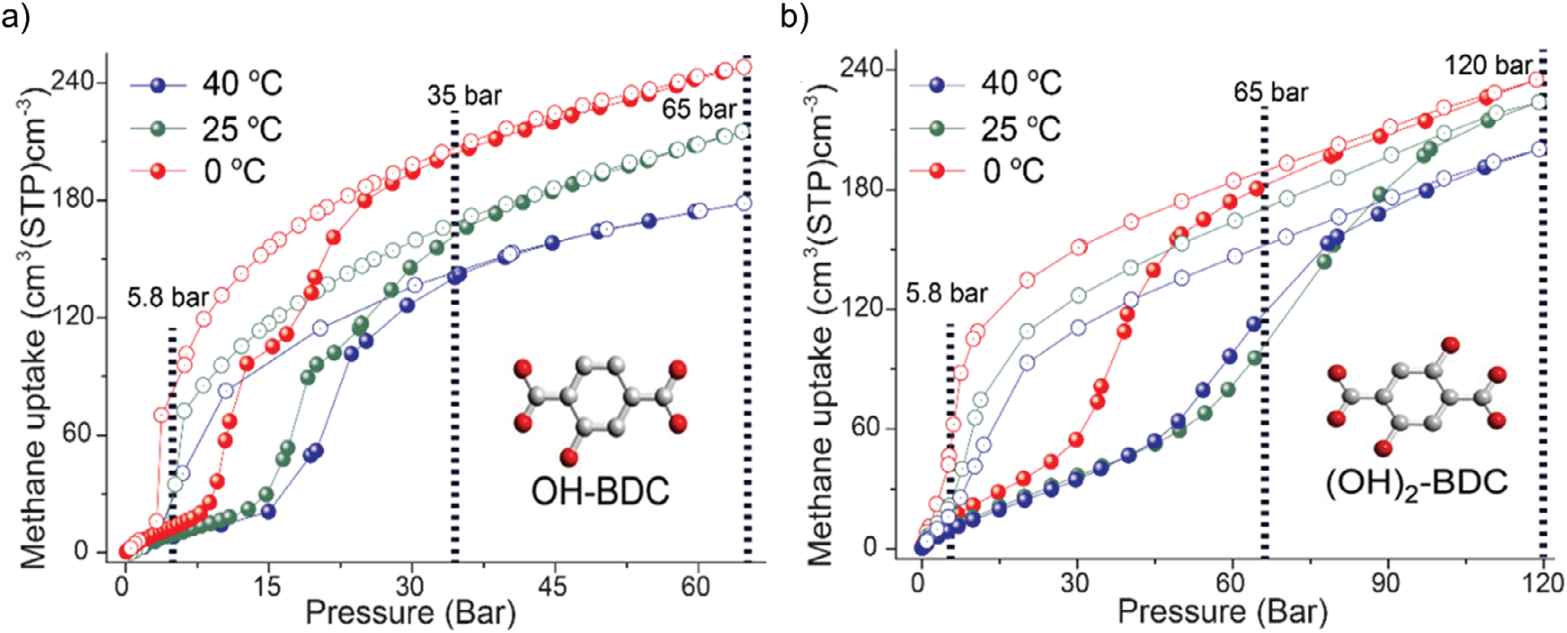
Comparison of gravimetric CH_4_ sorption isotherms at different temperatures for a) MIL‐53(Al)‐OH and b) MIL‐53(Al)‐(OH)_2_. Reproduced with permission.^[^ ^46^ ^]^ Copyright 2019, American Chemical Society.

Two isostructural flexible MOFs of the MIL‐53 series, namely, MIL‐53(Al)‐OH and MIL‐53(Al)‐(OH)_2,_ exhibit transitions from a narrow pore state to an open‐pore state during CH_4_ adsorption at 298 K, and the opening pressures are ≈15 and 46 bar, respectively, with hysteresis in the desorption isotherms leading to closure pressures of 4.1 and 5.8 bar^[^ ^46^ ^]^ (**Figure** **9**).

The higher transition pressure of MIL‐53(Al)‐(OH)_2_ is attributed to the additional ─OH groups, which enhance the intermolecular interactions and impede the transition of the framework (Figure 9a). At 298 K, the usable capacities for MIL‐53(Al)‐OH and MIL‐53(Al)‐(OH)_2_ in the 5–65 bar range are 71 and 164 cm^3^ cm^−3^, respectively.

The examples demonstrate the importance of the control over phase transition pressure at desired thermodynamic conditions. Such control can be realized by the constitutional changes of the building blocks (metal in the cluster or substituent on the linker), by the combination of multiple linkers in the same framework (solid solution),^[^ ^47^ ^]^ or by the adjustment of particle size.^[^ ^48^ ^]^

**Figure 10:**
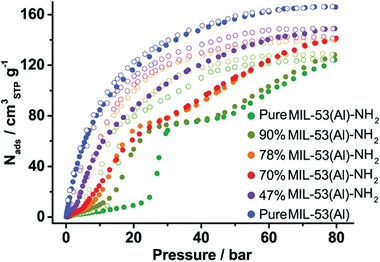
CH_4_ excess uptakes of MIL‐53(Al)–NH_2_, MIL‐53(Al), and MIL‐53(Al) with the BDC/BDC–NH_2_ mixed ligand at 298 K (filled symbols ‐ adsorption; empty symbols ‐ desorption). Reproduced with permition.^[^ ^47^ ^]^ Copyright 2017, Royal Society of Chemistry.

The mixed ligand strategy was demonstrated by Bolinois et al. in MIL‐53(Al), where the bdc was combined with NH_2_‐bdc in various ratios to induce flexibility upon high‐pressure methane adsorption (**Figure** **10**).^[^ ^47b^ ^]^

**Figure 11:**
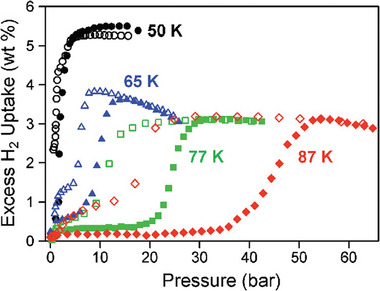
Isotherms for the excess uptake of H_2_ on [Co(bdp)]*_n_*, showing temperature‐dependent hysteresis loops at 50, 65, 77, and 87 K. Filled and open symbols represent adsorption and desorption curves, respectively. Reproduced with permission.^[^ ^53^ ^]^ Copyright 2008, American Chemical Society.

Very recently, Zhai group was able to show that by introducing a functional group (in this case, azido‐group), a formerly rigid CAU‐10 ([Al(OH)(1,3‐bdc)]*_n_*, 1,3‐bdc = isophthalate) framework^[^ ^49^ ^]^ can be transformed into a flexible one.^[^ ^50^ ^]^

So far, no MOF structure, rigid or flexible, has been able to meet the DOE's current targets for viable methane on‐board storage systems in terms of deliverable capacity. Flexible MOFs with a potential for high deliverable capacity are a promising class of materials that need further improvement in the pore volume and pore size optimization.

##### Hydrogen Storage in Flexible MOFs

Hydrogen is widely accepted as an environmentally friendly and promising alternative energy source. However, its extremely low critical temperature and standard boiling point (33 and 20 K, respectively) pose significant challenges for its storage by liquefaction or compression. Even under high pressures and low temperatures, H_2_ has a relatively low energy density per unit volume, making storage challenging.

DOE has set the gravimetric and volumetric working capacity targets of 5.5 wt%; 40 g L^−1^ for 2025, and 6.5 wt%; 50 g L^−1^ as an ultimate goal.^[^ ^51^ ^]^

Although several MOFs have high gravimetric working capacity reaching the DOE target, simultaneously high volumetric working capacity is still very challenging, and the current record holder (NPF‐200) has a working capacity of 37.2 g L^−1^ between 100 and 5 bar at 77 K.^[^ ^52^ ^]^

To the best of our knowledge, only a small number of MOFs have been reported where hydrogen adsorption induces gating due to framework flexibility at 77 K.

In 2008, Long and co‐workers reported the profiles of hydrogen high‐pressure physisorption isotherms of [Co(bdp)]*_n_* between 50 and 87 K (**Figure** 11).^[^ ^53^ ^]^ At 77 K and 40 bar, the excess adsorption capacity amounts to 3 wt%, and the corresponding gate opening and closing pressures are ≈20 and 10 bar. Unfortunately, the authors reported only excess H_2_ uptake values in their work and the total adsorption and working capacities were not calculated.

**Figure 12:**
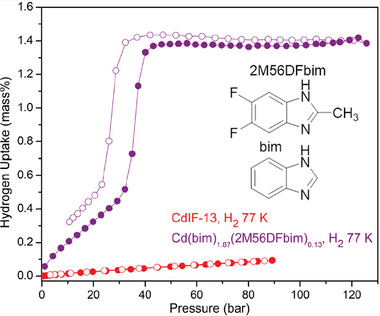
H_2_ physisorption isotherms at 77 K for CdIF‐13 (red) and [Cd(bim)_1.87_(2M56DFbim)_0.13]_ *_n_* (violet), exhibiting the reduced gate opening pressure threshold for the multivariate MOF whereas CdIF‐13 does not show any gate opening in this operating pressure regime. Closed circles correspond to adsorption, and open circles correspond to desorption. Reproduced with permission.^[^ ^54^ ^]^ Copyright 2023, American Chemical Society.

Recently, McGuirk and co‐workers^[^ ^54^ ^]^ reported a mixed linker approach successfully induced flexibility in [Cd(benzimidazolate)_2_]*_n_* (CdIF‐13) framework, which shows negligible uptake at 77 K in the pressure range up to 100 bar (**Figure** **12**). The 13% of 2‐methyl‐5,6‐difluorobenzimidazolate (2M56DFbim) instead of benzimidazolate (b induces gate‐opening for H₂. This stepped sorption profile enables a usable H₂ capacity of 1.17 wt%, which is, however, far below that of [Co(bdp)]*_n_*.

**Figure 13:**
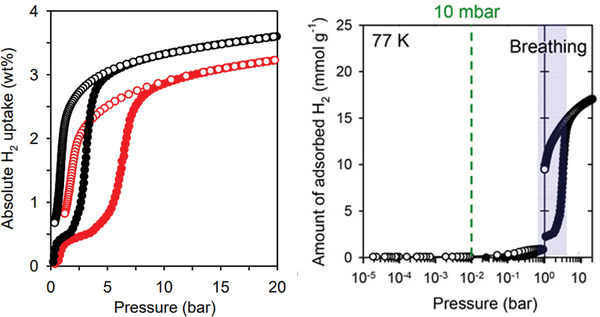
High‐pressure H_2_ physisorption isotherm of MIL‐53(Al) measured at 77 K (black) and 87 K (red). Reproduced with permission.^[^ ^55a^ ^]^ Copyright 2017, American Chemical Society.

MIL‐53(Al) was demonstrated to show gate opening during hydrogen adsorption at 77 and 87 K.^[^ ^55^ ^]^ Under vacuum and cryogenic conditions, the closed pore form is thermodynamically stable for desolvated MIL‐53(Al), resulting in no H_2_ adsorption in the low‐pressure range. According to the isotherm shape, the opening of the structure and the transition to the intermediate pore phase occurs at the pressure of ≈1 bar, followed by the opening transition at ≈3 bar at 77 K (**Figure** **13**).

Thus, in the case of H_2_ storage, DOI‐recommended values have not been achieved to date. However, the examples reported so far demonstrate that this area of research holds significant promise and is a highly encouraging direction for future exploration.

##### Acetylene Storage

Acetylene (C_2_H_2_) is a crucial raw material in industrial manufacturing and precision electronics. It is typically stored in acetone, as storing it under pressures exceeding 1.5 bar can lead to polymerization and potential explosion hazards.^[^ ^56^ ^]^ However, the presence of acetone vapours poses a challenge for applications that demand high‐purity C_2_H_2_.^[^ ^57^ ^]^ Therefore, there is a need for a more suitable storage medium for C_2_H_2_. Although extensive research has been conducted on C_2_H_2_ adsorption, most studies focus on atmospheric pressure, and no materials for high‐pressure storage have been reported. For flexible adsorbents, the inflexion point of the isotherm typically appears well below 1.0 bar. Nevertheless, even under low‐pressure adsorption conditions, it is possible to identify highly effective adsorbent materials. For instance, Kitagawa and co‐workers^[^ ^58^ ^]^ utilized synchrotron X‐ray powder diffraction data to determine the structure of a [Cu_2_(pzdc)_2_(pyz)]*_n_* (pzdc = pyrazine‐2,3‐dicarboxylate, pyz = pyrazine) MOF that adsorbs C_2_H_2_ molecules, discovering that C_2_H_2_ adsorption can readily induce significant framework distortion. This compound exhibits a high adsorption affinity for C_2_H_2_ (adsorption enthalpy 42.5 kJ mol^−1^), with an adsorption capacity in saturation of 42 cm^3^ g^−1^.

**Figure 14:**
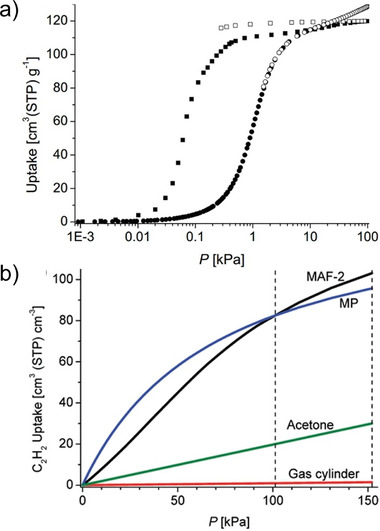
a) Semilogarithmic physisorption isotherms of C_2_H_2_ (squares) and CO_2_ (circles) for MAF‐2 at 195 K. The filled and open symbols represent adsorption and desorption, respectively. b) Volumetric C_2_H_2_ uptakes of MAF‐2 and other typical materials at room temperature. Two dashed lines represent the practical working limit of charging and discharging pressures. The curve marked as MP is shown for a hypothetical microporous adsorbent (following Langmuir isotherm) having the same uptake as MAF‐2 at 1 atm and *p*_0_. Reproduced with permission.^[^ ^57^ ^]^ Copyright 2009, American Chemical Society.

In 2009, Zhang et al. reported MAF‐2 ([Cu(etz)]*_n_*, Hetz = 3,5‐diethyl‐1,2,4‐triazole) MOF, which exhibited a sigmoid adsorption isotherm for C_2_H_2_. This facilitated the release of C_2_H_2_ in MAF‐2 and increased its usable capacity. The single‐crystal structure of C_2_H_2_‐loaded MAF‐2 revealed the formation of C_2_H_2_ hexamers within the pores. At 298 K and pressures between 1.0 and 1.5 bar, the estimated C_2_H_2_ uptake in MAF‐2 reaches 17 cm^3^ g^−1^ (equivalent to 20 cm^3^ cm^−3^), which is over 40 times the usable capacity of an equal volume gas cylinder (0.5 cm^3^ cm^−3^) (**Figure** **14**).

Subsequently, the same research group reported that MAF‐123‐Cd^[^ ^59^ ^]^ exhibited even higher working capacity. At 298 K, the C_2_H_2_ isotherm was nearly linear, with an uptake of only 2.23 mmol g^−1^ (54.514 cm^3^ g^−1^ or 83.55 cm^3^ cm^−^^3^) at 1 bar. However, at 273 K, the isotherm displayed a clear S‐shape, reaching an uptake of 6.34 mmol g^−1^ (142.06 cm^3^ g^−1^ or 217.85 cm^3^ cm^−^^3^) at 1 bar, indicating that a substantial usable capacity could be achieved through temperature swing adsorption. Based on the extrapolated isotherm (1.0–1.5 bar), the usable storage capacity of MAF‐123‐Cd was calculated to be 1.3 mmol g^−1^ (31.79 cm^3^ g^−1^ or 48.73 cm^3^ cm^−3^), which is 98 times higher than that of a standard gas cylinder (0.5 cm^3^ cm^−^^3^).

**Figure 15:**
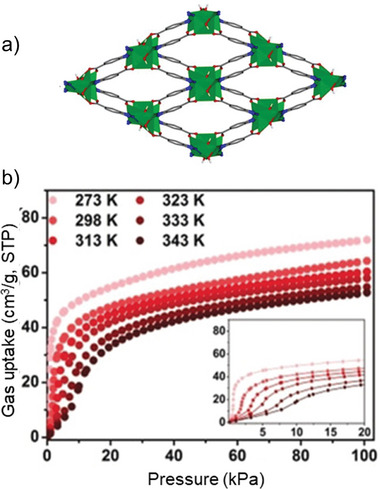
a) Structure of JNU‐1. b) C_2_H_2_ adsorption isotherms at different temperatures. Reproduced with permission.^[^ ^60^ ^]^ Copyright 2019, Wiley‐VCH GmbH & Co. KGaA.

In 2019, Zeng et al.^[^ ^60^ ^]^ investigated C_2_H_2_ adsorption on flexible microporous JNU‐1 ([Zn_3_(OH)_2_(btca)_2_]*_n_* btca = benzotriazole‐5‐carboxylate) MOF, possessing a high density of open metal sites (**Figure** **15**a). The isotherms display adsorption steps at low pressures that gradually disappear with the increase in temperature. Structural contraction of the pores upon the adsorption and induced‐fit effect leads to a significant rise in adsorption enthalpy. The binding between C_2_H_2_ and the MOF is exceptionally strong, making complete desorption possible only under high vacuum and elevated temperatures (Figure 15b). Therefore, in MOF design, it is crucial to balance binding affinity with regeneration energy, which involves managing the trade‐off between thermal management and the final usable capacity.

Moreover, flexible MOFs are highly likely to exhibit remarkable potential for storing highly toxic gases (such as AsH_3_, BF_3_, and PH_3_), which are critically important in the semiconductor industry. Currently, these gases are stored in cylinders, requiring extremely high levels of sealing. Despite the associated risks, the necessity of using such highly toxic gases highlights the significant application value of flexible adsorbent materials.

### 2.2 Mixed Gas Adsorption and Separation Concept

Separation and purification technologies account for up to 15% of global energy consumption and nearly half of industrial energy usage. Conventional gas separation technologies, such as distillation, involve repeatedly evaporating and condensing mixtures under harsh conditions. At the same time, liquid absorbents require heating and cooling large solvent volumes to release absorbed gases during regeneration. To reduce CO_2_ emissions, for example, methods like wet scrubbing of exhaust gases have been applied in industry for many years.^[^ ^61^ ^]^ The exhaust gases are led through an absorber filled with amine solution, which is capable of reacting with CO_2_ and forming carbamates. These processes are connected to high energy consumption, as the recovery of absorbents is only possible through thermal desorption. Hence, more energy‐efficient and sustainable gas separation processes are needed, and different methods and adsorbents have to be developed.^[^ ^62^ ^]^

One of the most important separation problems at present is the separation of CO_2_ from other gases, making selective CO_2_ adsorption from diverse gas mixtures an important research field.^[^ ^63^ ^]^ CO_2_ separation from an exhaust gas stream is one of the most challenging questions of the carbon capture process. The separation of these two gases in MOFs is proposed as an alternative to conventional materials.^[^ ^63^, ^64^ ^]^ Thus, it has been shown that MOFs would be able to significantly reduce energy consumption during regeneration, as the desorption of physisorbed CO_2_ requires less energy input as compared to recovery from carbamates. Comparing the so‐called parasitic energy, which is the energy penalty that is used to regenerate an adsorber or scrubber after sorption, it becomes clear that MOF adsorbents possess a huge advantage in this prospect.^[^ ^65^ ^]^ State‐of‐the‐art amine scrubbing technologies (with monoethanol amine; MEA) showed a parasitic energy higher than 1000 kJ kg^−1^, whereas rigid MOFs such as Mg‐MOF‐74 or Ni‐(4PyC)_2_ show much lower values of 727 and 655 kJ kg^−1^, respectively.^[^ ^65^, ^66^ ^]^

The important indicators for efficient adsorptive gas separation include also the working capacities of each component in a mixture, enthalpies or heats of adsorption, but also selectivities and mass transfer (adsorption kinetic).^[^ ^67^ ^]^ Despite extensive research in the field of MOF‐based gas separation/purification in more than the last two decades, the trade‐off of adsorption capacity versus selectivity is still a major challenge.^[^ ^68^ ^]^

**Figure 16:**
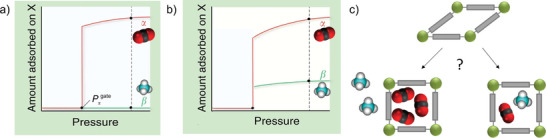
a–c) A key scientific question regarding mixed gas adsorption on flexible MOFs: How do the mixed gas adsorption isotherms for individual components behave in the pressure range above the phase transition pressure? a) Only one component (fluid triggering the transition) is adsorbed; and b) both components are coadsorbed, following the adsorption run of a prototypical rigid MOF with the same pore size and composition. c) Schematic illustration of two possible scenarios. Adapted with permission.^[^ ^22^ ^]^ used under CC BY 4.0. Copyright 2020, The authors.

The main advantage of flexible MOFs is the selectivity of the structural transitions against the triggering fluid. There are many examples of MOFs and fluids where the structural transition can be triggered by a specific fluid only at the specific conditions (**Figure** **16**b), or the pressure needed to provoke structural transition differs for various fluids.^[^ ^10^, ^69^ ^]^ In the case of MOFs with “gate opening” behavior, such phenomena could lead to incredibly high (near infinite) separation selectivity if such behavior could be translated to the case of a mixture of fluids (Figure 16a), and some of them have been introduced as “perfect” adsorbents.^[^ ^69^ ^]^

It should also be pointed out that even in a mixture, in many cases, a relatively high concentration of the “molecular opener” (i.e., the guest stimulating the phase transition) is required, implying that trace impurity separations based on flexible MOFs are less likely achievable.

The main concern, however, for flexible MOFs is whether the structural transition would suppress or enable the coadsorption of multiple components from the mixture (Figure 16), and whether the separation factors predicted from the single component isotherms can be transferred to the case of multicomponent adsorption.

A visionary goal would be the development of switchable MOFs in which the pores are accessible for one molecular species only (able to provoke structural transition and enter the pores) while the others in the mixture are kept outside (due to the unpreferable free energy of the system containing multiple components) (Figure 16a).

However, predicting the separation performance of flexible MOFs based solely on single‐gas physisorption isotherms is challenging, as both scenarios—exclusion of a second component or coadsorption—are theoretically possible, depending on the free energy profile of the MOF/adsorbate system. As a result, multicomponent adsorption measurements are the primary method for accurately evaluating the true separation capability of flexible MOFs.

To evaluate the separation performance of an adsorbent, the adsorption capacities for the fluids of interest are usually explored first. The most common method for measuring the adsorption capacity of MOFs for various gases is the static adsorption method. Here, the amount of gas adsorbed is determined in a state of equilibrium by adding a defined amount of gas to the sample. Once equilibrium has been reached, the amount adsorbed is determined either by the change in gas pressure (volumetric) or by the increase in sample mass (gravimetric).^[^ ^70^ ^]^ This method can also be used for gas mixtures, but in this case, the composition of the gas should be controlled beside the pressure change, and direct measurement of binary mixture equilibria is more complicated and time‐consuming.^[^ ^71^ ^]^

The researchers are still working to improve mathematical models for predicting mixture adsorption. Indeed, from the adsorption isotherms of individual gases, a prediction can be made using ideal adsorbed solution theory (IAST) or Grand Canonical Monte Carlo (GCMC) simulations.^[^ ^72^ ^]^ However, even for rigid adsorbents, the IAST cannot give information on deviations from ideal behavior.^[^ ^73^ ^]^

The calculations for flexible materials were not feasible for a long time since the contraction and expansion of the network could not be taken into account in these calculations, and the applied models were, therefore, invalid or subjected to significant errors.^[^ ^72^, ^74^ ^]^ In the past years, however, new methods, like the coupling of adsorbed solution theories and the thermodynamic osmotic ensemble, resulting in Osmotic Framework Adsorbed Solution Theory (OFAST)^[^ ^75^ ^]^ or hybrid Monte Carlo and Molecular Dynamics^[^ ^76^ ^]^ simulations, have been developed to come up with more accurate predictions.^[^ ^76^, ^77^ ^]^ The OFAST allows the prediction of phase transition pressures upon coadsorption, but its major drawback is that it relies on the IAST to describe adsorption in each phase of the host material.^[^ ^78^ ^]^ Thus, true selectivity is not accessible by this model if the adsorption behavior deviates from that expected by IAST.

It should also be noted that the OFAST model only deals with the thermodynamic stability of the phases of the material at equilibrium and yields no insight into the hystereses that are typically observed experimentally. Therefore, the theoretical predictions need to be verified by experimental data, leaving the most reliable method for determining sorption properties measuring the mixed gas adsorption isotherms.^[^ ^71^ ^]^

Breakthrough experiments can be considered as essential characterization technique for industrially relevant separation processes. Under dynamic conditions, many new parameters have to be considered to fully assert the suitability so that dynamic gas sorption measurements become necessary.^[^ ^70^, ^79^ ^]^ A breakthrough curve is a plot that reflects the concentration change of the adsorptive in the effluent stream at the outlet of a fixed bed adsorber, giving information not only about the adsorption capacity of the adsorbent but also about the kinetics of the adsorption processes.

Therefore, dynamic sorption experiments are one of the frequently employed methods for investigating gas separation problems. The behavior of flexible, phase‐changing adsorbents under dynamic breakthrough conditions can undergo several phase transitions, yielding nonconventional breakthrough profiles.^[^ ^80^ ^]^ The challenges for applications of flexible compounds in a bed are, amongst others, discussed below.

There are numerous highlights in the field of separation of diverse molecules on flexible MOFs, but due to limitations and the need to stay focused, we will limit ourselves to a few examples (such as some hydrocarbons, CO_2_/CH_4_ and D_2_/H_2_) here.

#### 2.2.1 Examples of Flexible MOFs for Separation

##### CO_2_/CH_4_

The carbon dioxide/methane separation ability of flexible MOFs is among the characteristics that have been heavily investigated. Significant differences in the adsorption enthalpies of the two gases render it likely that the framework will respond differently to each during the adsorption.

Mixed gas CO_2_/CH_4_ adsorption was intensively investigated experimentally and supported by theoretical calculations for MIL‐53(Cr) up to 25 bar at 303 K.^[^ ^81^ ^]^ Upon adsorption of pure CO_2_, the MOF undergoes a breathing‐type structural transition.

**Figure 17:**
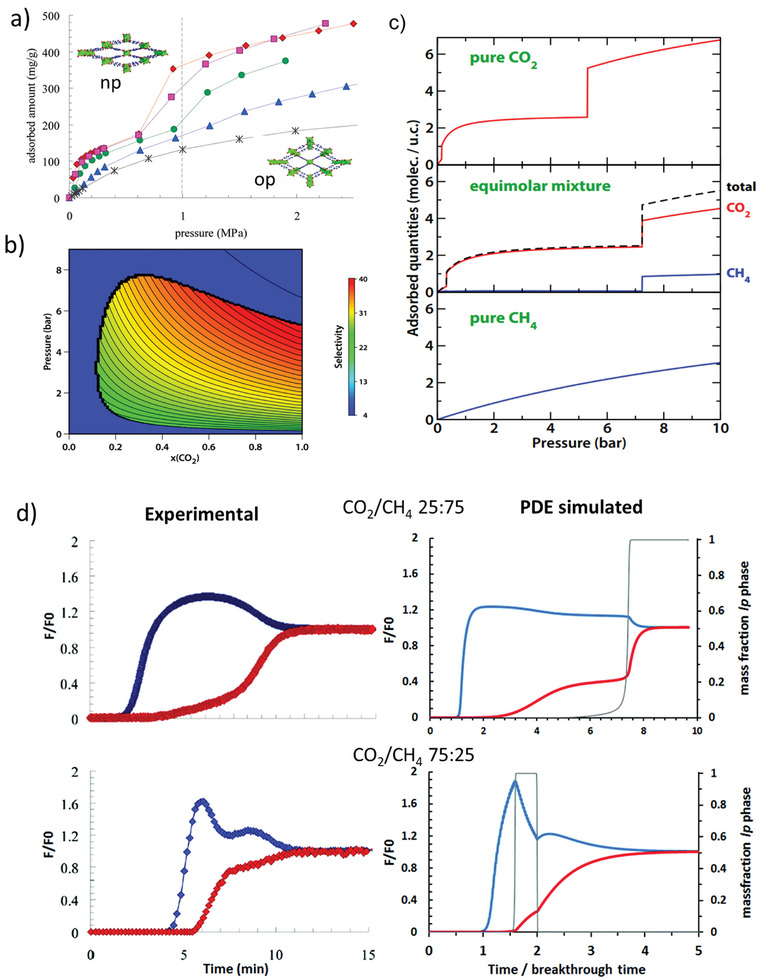
a) Total adsorbed amount of CO_2_/CH_4_ mixtures on MIL‐53(Cr) at 303 K. Red diamonds: pure CO_2_, pink squares: 75/25 CO_2_/CH_4_, green circles: 50/50 CO_2_/CH_4_, blue triangles: 25/75 CO_2_/CH_4_ and black crosses: pure CH_4_; Reproduced with permission.^[^ ^81^ ^]^ Copyright 2009, American Chemical Society. b) Calculated CO_2_/CH_4_ selectivity upon adsorption of a mixture in MIL‐53(Al), as a function of the total pressure and mixture composition. The narrow pore phase corresponds to the central “island,” with high selectivity, while the op phase has lower selectivity. Reproduced with permission.^[^ ^75^ ^]^ Copyright 2009, American Chemical Society. c) Calculated adsorption isotherms for a CO_2_/CH_4_ mixture in MIL‐53(Al) at 304 K as a function of pressure for pure CO_2_ (top panel), pure CH_4_ (bottom panel), and an equimolar mixture of the two (middle panel). Black dashed lines: total adsorbed quantity; red lines: quantity of CO_2_; blue lines: quantity of CH_4_. Reproduced with permission.^[^ ^75^ ^]^ Copyright 2009, American Chemical Society. d) Experimental breakthrough curve of binary CO_2_/CH_4_ mixtures on MIL‐53(Cr) at 303 K at 1.0 MPa (left). Reproduced with permission.^[^ ^81^ ^]^ Copyright 2009, American Chemical Society; and breakthrough profiles simulated by partial differential equations (PDEs). Reproduced with permission.^[^ ^80^ ^]^ Coryright 1016, Royal Society of Chemistry.

The adsorption of CH_4_ does not provoke structural transition in MIL‐53(Cr) in the investigated pressure and temperature range, showing the isotherm typical for microporous rigid adsorbent (**Figure** **17**a). The results of mixed gas adsorption experiments made clear that the composition of the employed gas mixture does have a substantial effect on the flexibility and capacity of the material. The total amount adsorbed was reduced with an increasing content of CH_4_ compared to the single gas adsorption of CO_2_ (Figure 17a). In situ Raman spectroscopic studies of the narrow pore and open phases revealed that both gases are coadsorbed, showing that there is a significantly higher affinity toward CO_2_, but in the presence of CH_4_, coadsorption is not completely hindered.^[^ ^81^ ^]^

For MIL‐53(Al), Coudert et al.^[^ ^75a^ ^]^ determined for each composition of the CO_2_/CH_4_ mixture whether breathing occurs and transition pressures of op to np as well as np to op phase by solving the OFAST equations numerically. Figure 17b demarcates the existence domains of the op (blue) and np forms (colorful) as well as selectivity in the (*x*(CO_2_), *p*) phase diagram of the mixture adsorption. The np phase can be seen as a high‐selectivity island (with values of selectivity in the range of a few tens), separated from the lower‐selectivity background that is the op phase. The pressure–composition phase diagram resulting from OFAST predictions is consistent with the experimental results of Finsy et al.,^[^ ^82^ ^]^ where two distinct selectivity mechanisms could be identified. Up to 5 bar at 303 K and equimolar gas mixture, the adsorption of CO_2_ is dominated by strong specific interactions with the np framework and average separation factors of ≈7. Above 6 bar, the average separation factor decreases to 4, due to the framework opening.

Denayer and co‐workers proposed a general methodology to model the behavior of flexible MOFs under dynamic breakthrough conditions.^[^ ^80^ ^]^ Each phase was modeled as a rigid adsorbent using a suitable adsorption model in correspondence to the method proposed by Coudert et al.^[^ ^83^ ^]^ The phase diagram was modeled by a sudden transition (SGE method) or a smoother s‐function (PDE method). The s‐function varies between 0 and 1 and can be physically correlated to the (mass)fractions of the occurring phases. The model was used to qualitatively simulate the breakthrough curves reported by Hamon et al.^[^ ^75^, ^81^ ^]^ The experimental and simulated F‐profiles are shown in Figure 17d. It was shown that the op to np transformation must be explicitly accounted. The feed conditions trigger the MIL‐53 to contract from the op to the np phase in equilibrium with the feed state, and the breakthrough profile shows an associated step. Simulations could capture a double roll‐up, noticed in the experimental breakthrough profile, and reveal the fractions of both adsorbent phases.

**Figure 18:**
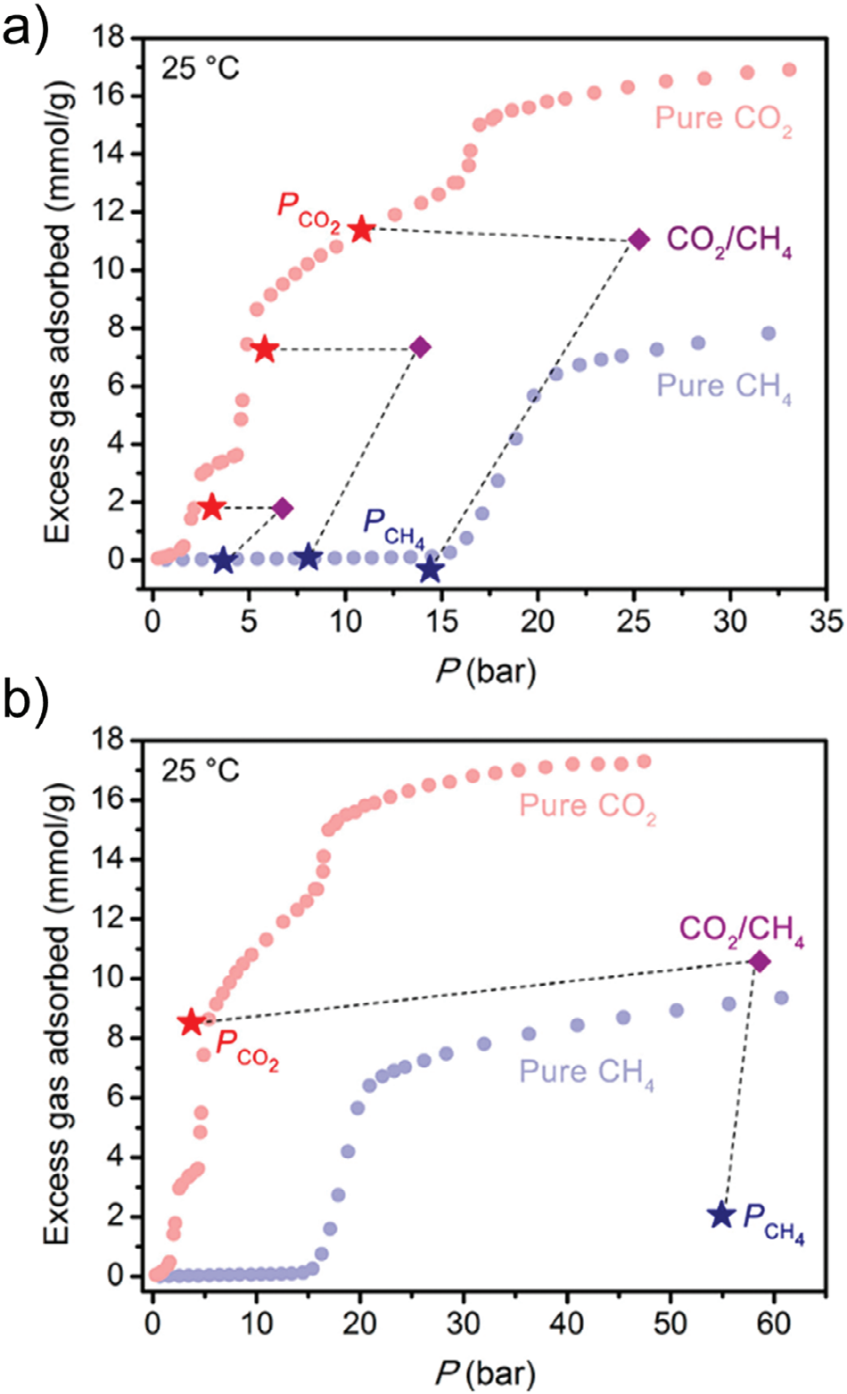
a) Multicomponent adsorption experiments for CO_2_/CH_4_ mixtures in [Co(bdp)]*_n_* show near‐perfect CO_2_ selectivity at 6.7, 13.9, and 25.3 bar, under equilibrium CO_2_/CH_4_ molar ratios of 46:54, 42:58, and 43:57, respectively. b) Multicomponent adsorption experiment performed under a CH_4_‐rich atmosphere (with an equilibrium CO_2_/CH_4_ molar ratio of 6:94) shows that [Co(bdp)]*_n_* adsorbs only a small amount of CH_4_ at this ratio, leading to a selectivity of 61 ± 4. (a,b) Purple diamonds represent the overall amount of gas adsorbed by [Co(bdp)]*_n_* (*y*‐axis) from a CO_2_/CH_4_ mixture at a given equilibrium pressure (*x*‐axis). Each purple diamond is paired with a corresponding red and blue star: red stars represent the CO_2_ adsorbed from the mixture (*y*‐axis) at the equilibrium partial pressure of CO_2_ (*x*‐axis), and blue stars represent the CH_4_ adsorbed from the mixture (*y*‐axis) at the equilibrium partial pressure of CH_4_ (*x*‐axis). Single‐component isotherms of CO_2_ (red circles) and CH_4_ (blue circles) are shown for reference. Reproduced with permission.^[^ ^69^ ^]^ Copyright 2018, American Chemical Society.

The selectivity for CO_2_ and CH_4_ mixture was investigated for [Co(bdp)]*_n_* MOF (Figure 5a).^[^ ^69^ ^]^ This MOF shows the gate opening behavior for both gases in the single‐component adsorption experiments, but the opening transition for CH_4_ occurs at a much higher pressure than that for CO_2_ (**Figure** **18**) and suggests that [Co(bdp)]*_n_* could be highly selective for CO_2_ at pressures between those corresponding phase transition pressures.

The composition of the adsorbate in the bicomponent CO_2_/CH_4_ (1:1) equilibrium adsorption experiment was monitored using mass spectrometry. The hypothesis based on the single‐component isotherms could be indeed confirmed, and [Co(bdp)]*_n_* adsorbs approximately no CH_4_ at the examined pressures and the framework is most accurately described as having near‐perfect CO_2_ selectivity under these conditions (Figure 18a). But, when the framework was exposed to a 6:94 molar ratio of CO_2_/CH_4_ at 58.6 bar (the region where the single component experiments suggest the opening for both gases), the material remained selective for CO_2_ (adsorbing 8.5 mmol g^−1^), but a significant amount of CH_4_ (2.1 mmol g^−1^) is also coadsorbed (Figure 18b).

**Figure 19:**
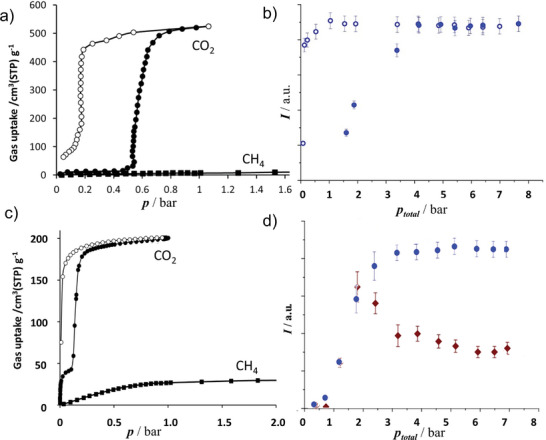
CO_2_ and CH_4_ single‐component physisorption isotherms for a) DUT‐8(Ni) and b) SNU‐9 measured volumetrically at 195 K. Signal intensities for adsorbed ^13^CO_2_ and ^13^CH_4_ measured by in situ ^13^C NMR spectroscopy for c) DUT‐8(Ni) and d) SNU‐9 pressurized with a ^13^CO_2_/^13^CH_4_ mixture (75:25) at 215 K. A full adsorption (filled symbols and desorption (empty symbols) cycle up to 7.6 bar is shown. No signal of adsorbed CH_4_ is detectable for DUT‐8(Ni). Reproduced with permission.^[^ ^43^ ^]^ Copyright 2019, American Chemical Society.

DUT‐8(Ni) ([Ni_2_(2,6‐ndc)_2_dabco]*_n_*, 2,6‐ndc = 2,6‐naphthalenedicarboxylate, dabco = 1,4‐diazabicyclo[2.2.2]octane), is an interesting and widely investigated model “gate opening” pillared layer MOF, showing selectivity in the gate opening for a large variety of molecular species in the gas and liquid states.^[^ ^10^, ^84^ ^]^ It has been shown that the network has a high affinity toward CO_2_ compared to other gases, including CH_4_, only showing the phase transition for CO_2_ at 298 K up to 60 bar.^[^ ^84b^ ^]^ To investigate the adsorption selectivity in the CO_2_ and CH_4_ mixture, the adsorption experiments were performed with the starting gas composition of 75:25 v/v at 215 K. The compositions of the adsorbed phase and gas phase were simultaneously monitored in situ by ^13^C NMR spectroscopy (**Figure** **19**a,b).^[^ ^43^ ^]^ To understand the influence of flexibility on the separation performance, comparable experiments were also conducted with the rigid version of DUT‐8(Ni). The flexible and rigid samples differ merely in the particle size. Analyzing the observed adsorbed and gaseous species of both gases in the resulting NMR‐spectra, it was proven that the flexibility of DUT‐8(Ni) causes nearly perfect selectivity, which is not the case for rigid MOF, showing significant uptake for both gases.

In situ ^13^C NMR spectroscopy was also applied to investigate the CO_2_/CH_4_ selectivity in the adsorption of a 75:25 mixture at 215 K for SNU‐9.^[^ ^43^ ^]^ It is capable of changing its pore structure between the np and op states during the adsorption of CO_2_ at 195 K through the formation of an intermediate (ip) phase. In contrast, CH_4_ does not initiate such transitions at 195 K, at least up to a pressure of 2 bar (Figure 19c).

Interestingly, the phase transition of SNU‐9 induces a significant change in adsorbed gas composition. Stepwise pressure increase upon adsorption results in a steeply increasing amount of adsorbed CO_2_ upon the structural np–ip–op transition. The absolute amount of methane coadsorbed with CO_2_ is relatively low in the np state. It increases in the ip state and decreases after the transition into the op state, thus passing through a maximum in the ip state (Figure 19d). At higher pressures, the NMR signal of adsorbed CH_4_ decreased in intensity while the gaseous CH_4_ signal increased, indicating the desorption of CH_4_. In contrast to that, no desorption of CO_2_ was observed, resulting in a selectivity varying strongly with the current phase of the MOF.

Schneemann et al. investigated the coadsorption on a series of substituted [Zn_2_(bdc)_2_(dabco)]*_n_* (D‐MOF) MOFs belonging to the pillared layer materials.^[^ ^18a^ ^]^ D‐MOF itself does not show pronounced flexibility upon adsorption of methane or carbon dioxide.

**Figure 20:**
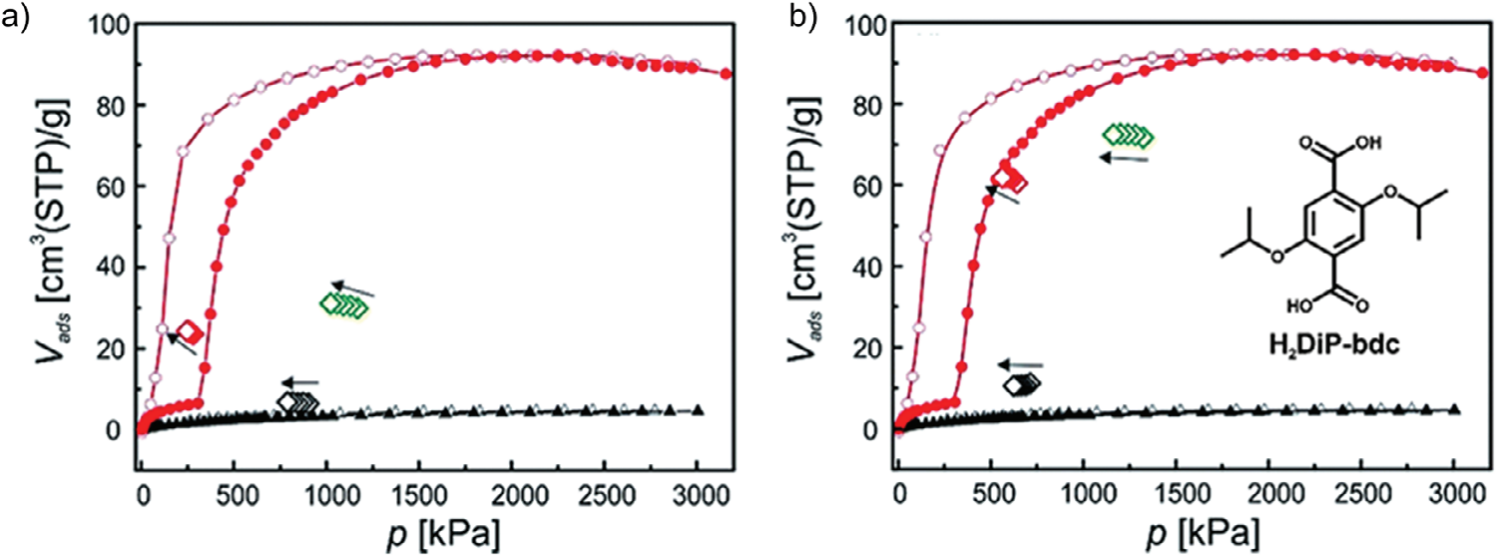
Depiction of the excess single component and coadsorption measurements for [Zn_2_(DiP‐bdc)_2_(dabco)]*_n_*: CO_2_ (red), CH_4_ (black), total (green). Reproduced with permission.^[^ ^18a^ ^]^ Copyright 2016, Royal Society of Chemistry.

By exchanging bdc by DiP‐bdc (2,5‐diisopropoxy‐1,4‐benzenedicarboxylate), however, flexibility could be unlocked. The single‐component isotherms show that the structural transition can be induced by CO_2_ only, up to 30 bar investigated in the experiment. In the first run of the coadsorption experiment, the partial CO_2_ pressure was chosen to be slightly below the np → op transition pressure and during the second experiment, the partial CO_2_ pressure was above the np → op transition pressure. From the first experiment (**Figure** **20**a), it could be seen that the adsorbed amount of CO_2_ during the coadsorption experiments is much higher than for the single component adsorption at the same pressure (23.47 compared to 6.5 cm^3^ g^−1^), while the CH_4_ uptake is only slightly increased (6.5 compared to 4.6 cm^3^ g^−1^), suggesting that a part of the sample has been already transformed to op state upon experiment. In the second experiment (Figure 20b), it can be clearly seen that the CO_2_ uptake coincides with the CO_2_ uptake from the single‐component experiment and the CH_4_ is coadsorbed.^[^ ^85^ ^]^

**Figure 21:**
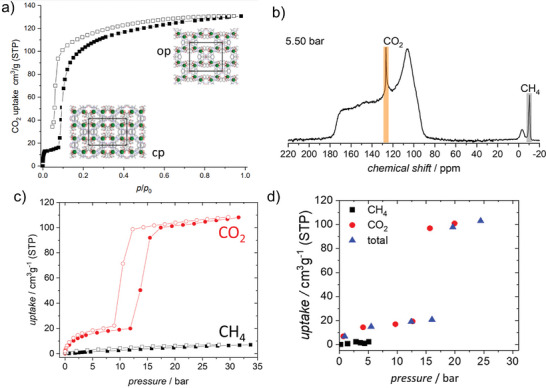
a) CO_2_ physisorption isotherm of JUK‐8 at 195 K and the crystal structures of corresponding cp and op phases. b) ^13^C NMR spectrum of the ^13^CO_2_/^13^CH_4_ gas mixture at 5.5 bar and 195 K. The signal at −10 ppm (grey bar) corresponds to gaseous methane, at −4 ppm to the adsorbed methane, at 127 ppm to the gaseous carbon dioxide (orange bar), and the broad signal between 90 and 180 ppm to the adsorbed carbon dioxide. c) Single component methane and carbon dioxide physisorption isotherms at 298 K. d) Isothermal multicomponent adsorption experiments for CO_2_/CH_4_ (75:25 v/v) mixtures at 293 K. Reproduced with permission.^[^ ^86^ ^]^ Copyright 2021, The Authors. Published by American Chemical Society. This publication is licensed under CC‐BY 4.0.

The adsorption of CO_2_/CH_4_ mixture on JUK‐8 (([Zn(oba)(pip)]*_n_*, oba = 4,4′‐oxybis(benzenedicarboxylate), pip = 4‐pyridyl‐functionalized benzene‐1,3‐dicarbohydrazide) was intensively investigated by Roztocki et al. by a combination of different techniques.^[^ ^86^ ^]^ JUK‐8 possesses a gating type of flexibility, where the structural transition from cp to op phase is induced by CO_2_ (**Figure** **21**a). In situ ^13^C NMR studies at 195 K point on the coadsorption of both gases in the open pore phase at 5 bar for 1:1 CO_2_/CH_4_ ratio. At temperatures above 280 K, however, the near‐perfect selectivity is achieved, and only CO_2_ seems to be adsorbed from a CO_2_/CH_4_ (75:25 v/v) mixture.

**Figure 22:**
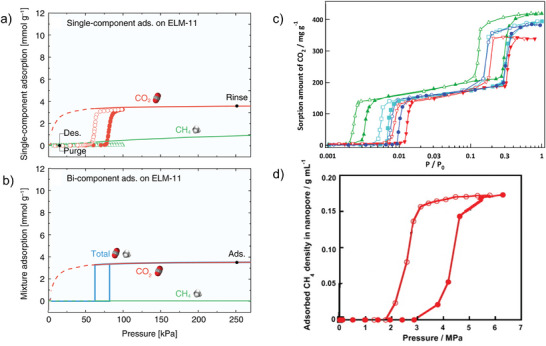
a) Single‐component adsorption isotherms of CO_2_ and CH_4_ at 298 K on ELM‐11 (filled symbols: experimental adsorption data, open symbols: experimental desorption data, lines: adsorption data simulated by GCMC). b) Total and component adsorption isotherms of an equimolar mixture of CO_2_ and CH_4_ at 298 K for ELM‐11 simulated by GCMC. The abscissae correspond to the partial pressures of CO_2_.Reproduced with permition.^[^ ^22^ ^]^ Copyright 2020, The Authors, Published by Springer Nature Limited under CC BY 4.0. c) CO_2_ sorption isotherms of ELM‐11 measured at different temperatures. Green, 195 K; light blue, 253 K (*p*_0_ = 1.96 MPa); blue, 273 K (*p*_0_ = 3.47 MPa); red, 298 K (*p*_0_ = 6.4 MPa). Open and filled symbols indicate sorption and desorption branches, respectively, in each successive run. Reproduced with permission.^[^ ^87^ ^]^ Copyright 2016, American Chemical Society. d) CH_4_ adsorption/desorption isotherm of ELM‐11 at 303 K. Adapted with permission.^[^ ^34^ ^]^ Copyright 2009, Elsevier Inc.

Recently, CO_2_/CH_4_^[^ ^22^ ^]^ separation was extensively investigated for gate opening ELM‐11 by Hiraide et al. for application in pressure vacuum swing adsorption systems. The CO_2_ adsorption isotherm at 298 K shows two steps. The first step (cp to np transition) occurs at the relative pressure of 10^−3^ (0.75 bar). The second step (np to op transition) was observed at *p*/*p*_0_ ≈ 0.3 (19.2 bar) (**Figure** **22**c). Methane adsorption isotherm of ELM‐11 at 303 K indicates the opening transition at ≈40 bar and closing transition upon the desorption is finished at ≈20 bar).^[^ ^34^ ^]^ Thus, the opening of the cp phase to the np phase can be provoked by carbon dioxide but not by methane, and the operation window between 1 and 19 bar should guarantee high selectivity (Figure 22). At pressures above 20 bar, the compound is expected to adsorb both components.

**Figure 23:**
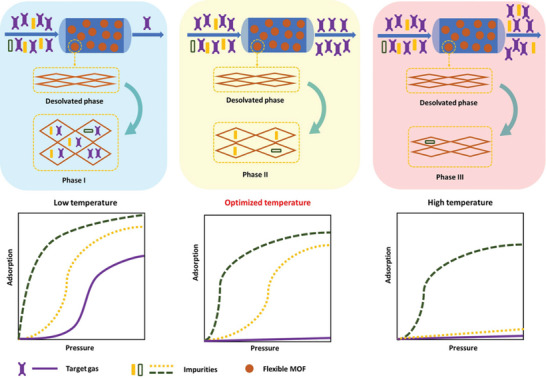
Illustration of the temperature optimization for ternary mixture separation by flexible MOFs. At low temperatures, all three gases can be adsorbed, which leads to the coadsorption of target gas and low productivity. At optimized temperature, two impurities are selectively adsorbed, and pure target gas is obtained in high yield. At high temperatures, only one type of impurity is adsorbed, therefore, the product is not pure. Reproduced with permission.^[^ ^88^ ^]^ Copyright 2020, Wiley‐VCH GmbH.

To tune the separation performance of flexible MOFs, the building blocks of the MOF can be adjusted, such as metal in the cluster or substituent on the linker, as discussed above, but also the temperature can be adapted since the temperature belongs to important thermodynamic parameters and strongly influences the free energy landscape of the given system.^[^ ^22^, ^37^ ^]^ In 2020, Dong et al. demonstrated that the separation performance can be optimized through tuning gate‐opening pressure by temperature (**Figure** **23**) on the example of ternary mixture separation in flexible NTU‐65 ([Cu(L1)_2_SiF_6_)]*_n_*_,_⋅L1 = 1,4‐di(1H‐imidazol‐1‐yl)benzene).^[^ ^88^ ^]^

The authors showed that by varying the adsorption temperature, the uptake was strongly influenced, allowing for high uptakes of CO_2_ and C_2_H_2_ but decreasing the amount adsorbed of C_2_H_4_ when optimized. Using the obtained information from static single gas adsorption, the separation of the ternary mixture (C_2_H_4_/C_2_H_2_/CO_2_ 90/1/9 v/v/v) was possible at the optimized temperature of 263 K yielding high purity >99.99 % for C_2_H_4_ after only one cycle.

##### Light Hydrocarbon Separation (C2–C3)

Light hydrocarbons are not only important energy resource but also important raw materials for fine chemicals. The similarity of boiling points, kinetic diameters, dipole moments, and other physical properties makes the separation and purification of light hydrocarbons challenging, making the applications of flexible MOFs very attractive.^[^ ^89^ ^]^

The adsorption of methane, ethane, propane, ethylene, and propylene was studied using ZIF‐7 ([Zn(bim)_2_]*_n_*, bim = benzimidazolate) at 298 K.^[^ ^90^ ^]^ Except for methane, all the gases show gating isotherms, with distinct differences between the gate opening and closing pressures, leaving a window of selective uptake operation. It could be shown in breakthrough experiments that ethane is selectively adsorbed over ethylene in their mixtures, which results in the direct production of pure ethylene.

It makes ZIF‐7 a perfect candidate for the separation of olefins from paraffins since, in contrast to most microporous materials, the paraffin is selectively adsorbed.

The potential of [Cu(dhbc)_2_(4,4′‐bipy)]*_n_* for C1–C3 hydrocarbon hydrocarbon separation was tested at 298 K.^[^ ^91^ ^]^ The single‐component isotherms of each C2–C3 hydrocarbon display gating and differ in gate opening pressures, which inversely correlate well with the latent heat of vaporization of the corresponding hydrocarbon. C_3_H_4_ has the lowest gate opening pressure, which indicates that the flexible framework can highly selectively adsorb C_3_H_4_ at a very low pressure. Several breakthrough experiments were performed using equimolar C_3_H_4_/C_3_H_6_/C_3_H_8_ mixtures at 298 K. C_3_H_6_ and C_3_H_8_ break first, while C_3_H_4_ breaks through after some period of time. C_3_H_4_ can be effectively separated from the C_3_H_4_/C_3_H_6_/C_3_H_8_ mixtures in a nearly pure form with gas phase concentrations of more than 99.9%.

[Co(vttf)]*_n_* (vttf = 2,2′‐[1,2‐bis(4‐benzoate)−1,2‐ethanediylidene]bis‐1,3‐benzodithiole)) reported by Kitagawa and co‐workers, exhibits exclusive gate opening for ethylene, potentially enabling the discriminatory adsorption of it over ethane.^[^ ^92^ ^]^ In the close pore phase, the compound is nonporous and features crosslinking via the coordination of tetrathiafulvalene sulfur atoms with the axial sites of the paddle wheels. The framework is not responsive to ethane, but the ethylene is able to coordinate cobalt and, therefore, induces a phase transition, displacing the tetrathiafulvalene linkers and yielding an open structure. Once open, however, the framework adsorbs both ethylene and ethane, resulting in only modest selectivities.

The adsorption ability of triply interpenetrated, flexible SD‐65 ([Zn(NO_2_ip)(dpe)]*_n_*, NO_2_ip = 5‐nitroisophthalate, dpe = 1,2‐di(4‐pyridyl)ethylene) was studied for seven C_4_ hydrocarbons, including 1,3‐butadiene, *trans*‐2‐butene, *cis*‐2‐butene, *n*‐butane, 1‐butene, isobutene, and isobutane at 298 K.^[^ ^93^ ^]^ The uptake of all butenes and butanes was negligible at 1 bar, but the 1,3‐butadiene shows a gate‐opening sorption profile with a gate‐opening pressure of 60 kPa, gate‐closing pressure of 50 kPa and 40 cm^3^ g^−1^ uptake at 101 kPa. Therefore, SD‐65 can separate 1,3‐butadiene from C4 hydrocarbon mixtures and readily release adsorbed 1,3‐butadiene under the PSA conditions.

##### H_2_/D_2_

Deuterium, constituting 0.016% of total hydrogen occurring in nature, is a scientifically and industrially relevant molecule, but its separation from H_2_ is challenging due to its similar physical properties.

**Figure 24:**
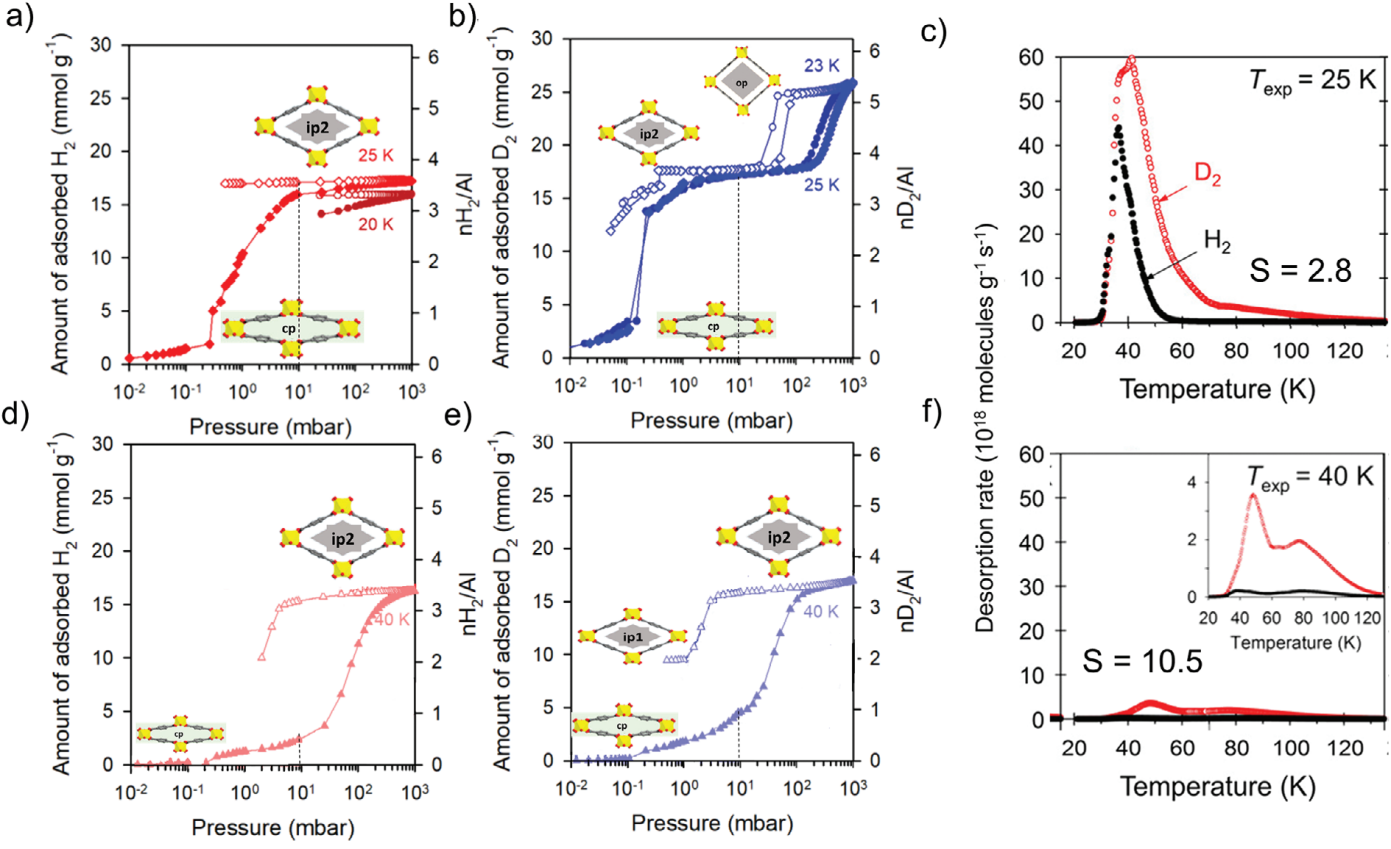
Hydrogen (red) and deuterium (blue) physisorption isotherms of MIL‐53(Al): a) H_2_ at 20 and 25 K, b) D_2_ at 23 and 25 K, c,d) H_2_ at 40 K, e) D_2_ at 40 K. (c,f) TDS of D_2_/H_2_ (1:1) mixed gas from MIL‐53(Al) exposed at 10 mbar. Reproduced with permission.^[^ ^55^ ^]^ Copyright 2017, American Chemical Society; Reproduced with permission.^[^ ^94^ ^]^ Copyright 2020, American Chemical Society.

Physisorption of H_2_ and D_2_ at different temperatures has been studied in detail for MIL‐53(Al) (**Figure** **24**).^[^ ^55^, ^94^ ^]^ The experiments show that the selectivity for D_2_ over H_2_ is strongly related to the state of the pore structure of MIL‐53(Al). At temperatures below ≈150 K, the solvent‐free MIL‐53(Al) forms the closed pore phase (unit cell volume *V* = 857 Å^3^), inaccessible for adsorptives.^[^ ^95^ ^]^ A two‐step transition is observed upon adsorption of D_2_ at the boiling point temperature, where the structure transforms from the cp to the intermediate pore phase (ip2, *V* = 1349 Å^3^) and further to the open pore phase (*V* = 1531 Å^3^) (Figure 24b). Upon hydrogen adsorption, however, the framework transforms only to the ip2 phase at the corresponding boiling point temperature (Figure 24a). During desorption, additional intermediate phases are formed, which is evident from the multiple steps in the desorption branch. In general, 130 different structures of the MIL‐53 family are deposited in the Cambridge Structural Database,^[^ ^96^ ^]^ showing the complexity of the free energy landscape of this compound.

The selectivity of H_2_/D_2_ adsorption was studied by thermal desorption spectroscopy (TDS), at which the MOF was subjected to the 10 mbar of 1:1 mixture at 25 K. The experiment shows no outstanding selectivity because both gases transform the MOF to the thermodynamically stable ip2 phase at these conditions.

Nevertheless, optimizing the conditions can lead to an increased selectivity of up to 10.5 at 10 mbar and 40 K. At these conditions, the breathing of the network is in progress, leading to the optimized environment for quantum sieving (Figure 24d–f).

**Figure 25:**
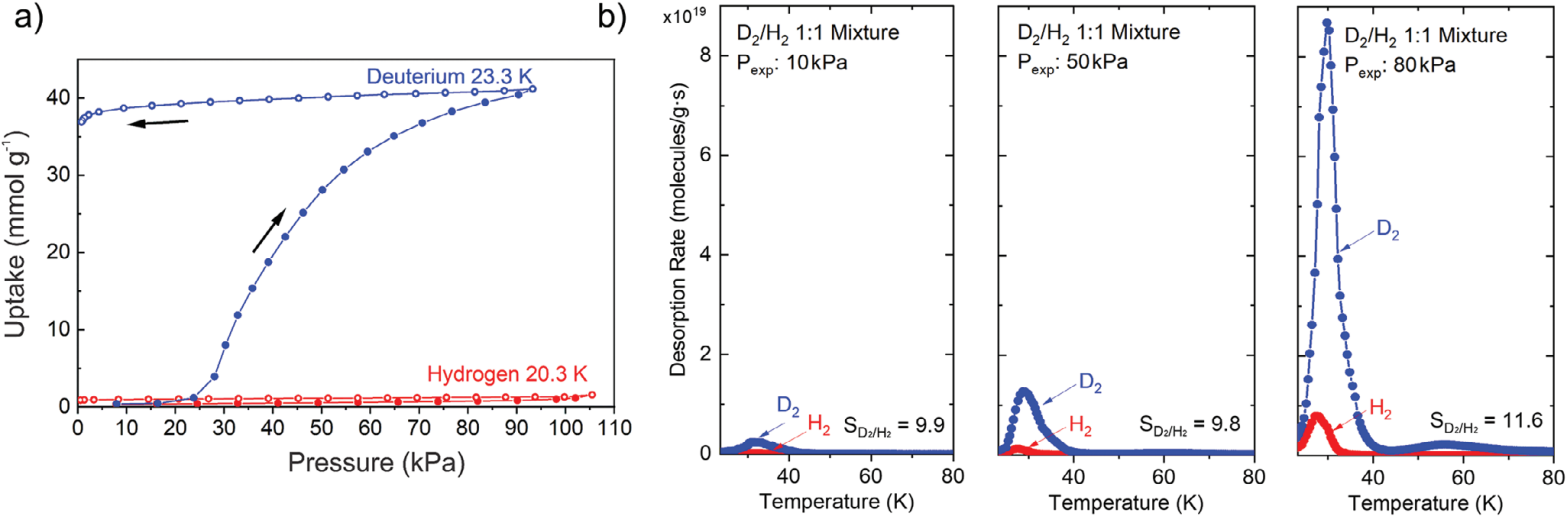
a) H_2_ and D_2_ isotherms for DUT‐8(Ni) at 20.3 and 23.3 K. b) TDS spectra for DUT‐8(Ni) and D_2_/H_2_ selectivities (S_D2/H2_) according to the exposed pressure. Measurements were performed under a H_2_/D_2_ (1:1) isotope mixture at 23.3 K. Reproduced with permission.^[^ ^97^ ^]^ Copyright 2022, The Authors, some rights reserved; exclusive licensee American Association for the Advancement of Science. Distributed under CC BY‐NC 4.0.

The ability to separate hydrogen isotopes was also investigated for the gate‐opening DUT‐8(Ni) (**Figure** **25**).^[^ ^97^ ^]^ Low‐temperature adsorption of H_2_ and D_2_ gas, each at their standard boiling points, reveals pressure‐dependent responsivity toward D_2_. The adsorbed amount of H_2_ is barely above the detection limit with a slight increase up to 1 mmol g^−1^ at 1 bar. Thus, DUT‐8(Ni) remains in the cp phase under the H_2_ atmosphere. In stark contrast, after little to no uptake at low pressure, there is steep adsorption of D_2_ at 0.24 bar, the gate‐opening pressure resulting in a huge saturation uptake of 41.2 mmol g^−1^ at 1 bar. The desorption branch indicates a hysteresis, as expected for a first‐order structural transition.

TDS after exposure of the sample to H_2_/D_2_ (1:1) isotope mixture shows that the D_2_ uptake is approximately ten times higher than for H_2,_ and the average selectivity is 9.9. The D_2_ uptake increases with increasing total pressure of the mixture to 0.8 bar and shows the highest value of 9.44 mmol g^−1^ together with the best selectivity of 11.6. Nevertheless, the experiments show that both gases are coadsorbed at the chosen conditions.

### 2.3 How to Maximize Selectivity in Flexible MOFs

Analyzing the experimental examples of various mixed adsorption experiments, we propose a hypothesis, answering the question posed in Figure 16: whether the structural flexibility would suppress or enable the coadsorption of multiple components from the mixture, what is the underlying reason, and what are the prerequisites for highest selectivity? Analyzing the cases reported, it can be seen that both scenarios are possible: near‐perfect selectivity as well as coadsorption of both components.

Obviously, the reason is hidden in the isotherms runs for the individual components, or more precisely, in the overlay of two isotherms and thermodynamic stability of the framework/gas_α/gas_β system at experimental condition chosen for coadsorption/separation experiments.

It is important to consider the hysteretic behavior of the isotherms when designing the separation processes. The hysteresis occurs due to the activation barriers separating the phases with energetic minima and energy penalty associated with the nucleation and increasing interfacial area upon the phase transformation.

The desorption branch of the gating isotherms is closer to thermodynamic equilibrium, while the adsorption branch reflects the system accessing metastable states. Thus, the kinetic barriers determine the opening pressure of the framework, while the closing pressure determines the thermodynamic stability of the framework filled with the adsorbate.

In the case of breathing, the contraction of the op phase to the np phase is characterized by a much lower barrier than the opening (cp to op, or np to op transitions upon the desorption). Thus, in this case, the adsorption branch upon the first breathing transition is closer to thermodynamic equilibrium, while the desorption branch reflects the system accessing metastable state. Thus, the kinetic barriers determine the expansion pressure of the framework, while the contraction pressure determines the thermodynamic stability of the framework filled with the adsorbate.

Below some particular cases are considered.

#### 2.3.1 Case I: Gate Pressure MOF

**Figure 26:**
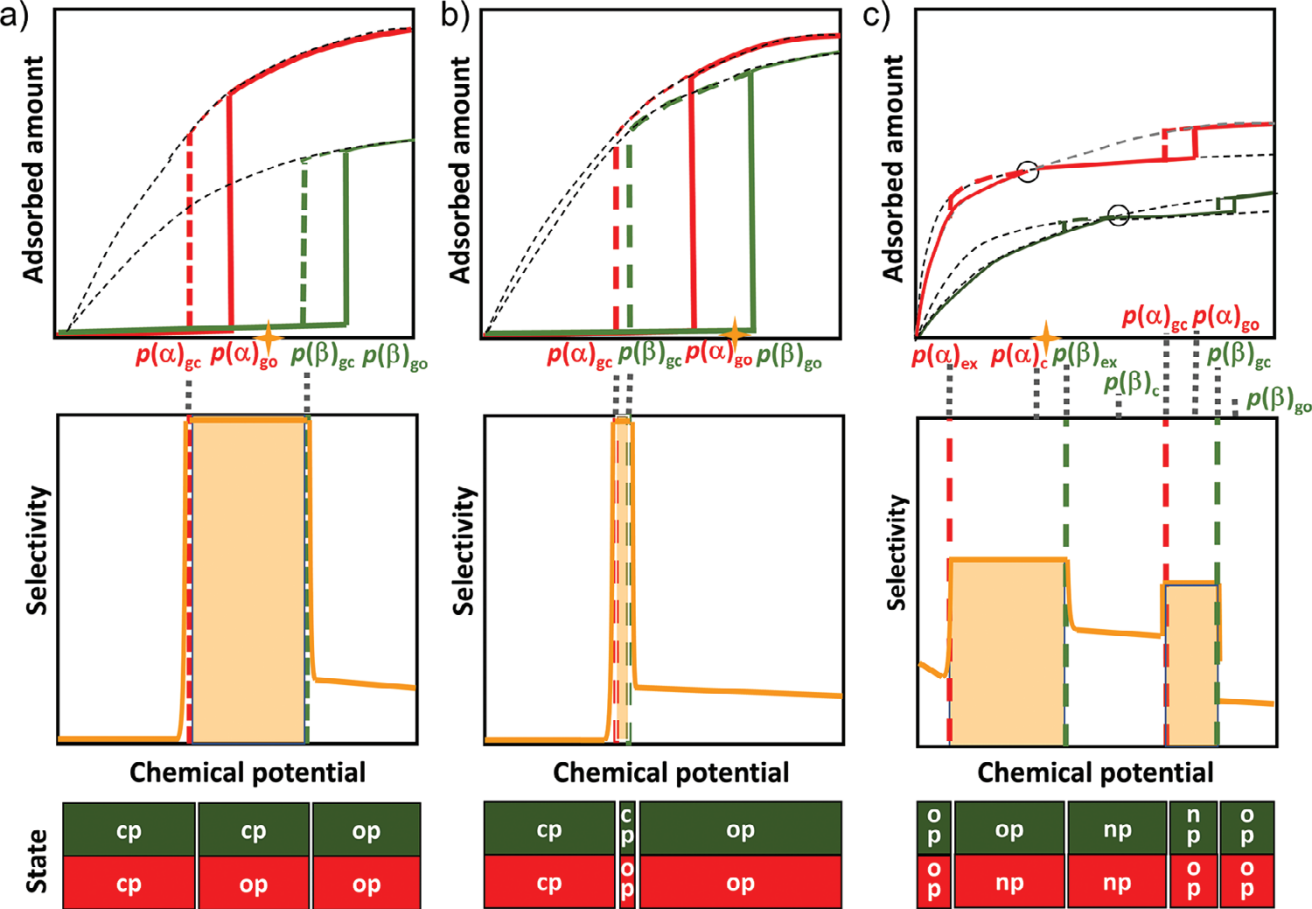
Hypothetical single component isotherms for a,b) hypothetical gate‐opening or c) breathing MOF (op to np transition) (upper row) and the expected selectivity in the case of an equimolar mixture of the components (middle row). The adsorption is shown as a solid, colored line, and the desorption is shown as a dashed line of the corresponding color. The grey dashed lines represent the isotherms of the hypothetical “rigid” MOFs with comparable porosity. The star marks one of the optimal operation pressures. The bottom row shows the thermodynamically stable state of the MOF in the presence of each gas in the corresponding chemical potential range.

The gate opening pressure (*p*_go_) for adsorptive α is lower than the gate closing pressure (*p*_gc_) for adsorptive β (**Figure** **26**a). In this case, three potential pressure ranges exist.

1. Below *p*(α)_go_, where the adsorbed amount is neglectable due to the thermodynamic (*p* < *p*(α)_gc_) or kinetic (*p*(α)_gc_ < *p* < *p*(α)_go_ stability of the cp phase.
2. Between *p*(α)_gc_ and *p*(β)_gc_ is the region of nearly perfect selectivity, where the free energy of the open phase is favorable only if the pores contain the adsorptive α. The partial pressure of component α in the experiment should, however, be larger than *p*(α)_go_ to initiate the structural transition. (For example, see the behavior of DUT‐8(Ni)^[^ ^43^ ^]^ (Figure 19a,b) or [Co(bdp)]*_n_* ^[^ ^69^ ^]^ (Figure 18a) in CO_2_/CH_4_ adsorption.)
3. Above *p*(β)_gc_, the selectivity is expected to drop to the value characteristic for the virtually rigid framework with the same pore size and chemistry. (For example, see the behavior of [Co(bdp)]*_n_* ^[^ ^69^ ^]^ (Figure 18b) in CO_2_/CH_4_ adsorption at low CO_2_ concentration.)

#### 2.3.2 Case II: Gate Pressure MOF

The gate closing pressure (*p*_gc_) for adsorptive β is lower than the gate opening pressure (*p*_go_) for adsorptive α (Figure 26b).

The pressure regions defining the adsorption selectivity are the same as in Case I, but the window of the highest selectivity is much narrower. Moreover, the pressure needed for the framework opening (exemplarily marked by the star in Figure 26b) is necessarily outside the selectivity window, making the separation not feasible. Thus, the adsorption selectivity is expected to be similar to that of a hypothetical rigid MOF, because the open pore phase at pressures above *p*(β)_gc_ is thermodynamically stable in the presence of both individual components. Case II is expected for high activation barriers (broad hysteresis) as well as for guests with similar adsorption enthalpies.

It should be mentioned that if the physisorption isotherms of the individual components are measured in the limited pressure range only (below *p*(β)_go_), the picture can pretend Case I, leading to a wrong assessment of the expected selectivity.

#### 2.3.3 Case III: Breathing MOF

The breathing transition usually involves two transformations: the contraction of the more porous phase in the presence of a certain amount of adsorbate and the expansion (gate opening) to the more porous phase at higher pressures upon adsorption. Upon desorption, the framework contracts to the np phase first and, at lower pressure, expands to the initial, guest‐free framework.

For simplicity, we only consider the case where the pressure of the first transition (contraction) at characteristic contraction pressure of α component (p(α)_c_), is much lower than the contraction pressure for the β component (*p*(β)_c_) and the pressures of the gate opening (*p*(α)_go_ and *p*(β)_go_ and closing (*p*(α)_gc_ and *p*(β)_gc_) in the second transitions (gating).

The activation barrier of the contraction transition is usually very low. The expansion upon the desorption, however, is kinetically hindered, resulting in the hysteresis between adsorption and desorption branches and *p*_ex_ < *p*_c_.

If the contraction pressure for adsorptive α (*p*(α)_c_) is lower than the expansion pressure for adsorptive β (*p*(β)_ex_), then (Figure 26c):

1. At pressures below (*p*(α)_ex_) the selectivity is approaching that of the op phase. (For example, see CO_2_/CH_4_ separation on MIL‐53(Al)^[^ ^75^ ^]^ at low pressure, Figure 17.)
2. In the pressure range between *p*(α)_ex_ and *p*(β)_ex_, where the narrow pore phase is thermodynamically stable only in the presence of adsorbate α, the preferable adsorption of component α is expected. (For example, see CO_2_/CH_4_ separation on MIL‐53(Al)^[^ ^75^, ^81^ ^]^ at moderate pressure, Figure 17.)
3. At pressures above (*p*(β)_ex_) and below *p*(α)_gc,_ np phase can be stabilized by each component, (α or β), therefore, the selectivity is approaching that of the virtual, rigid np phase.

If we would take into consideration the second gating phase transition of the breathing (the np to op transition), situations similar to that described in Cases I and II are feasible, as well as more complicated cases, depending on the values of *p*_c_(α), *p*_c_(β)*, p*_ex_(α), *p*_ex_(β)*, p*_go_(α), *p*_go_(β)*, p*_gc_(α), and *p*_gc_(β).

But considering the fact, that in the breathing system, the closed pore phase (unporous state) does not exist, the maximum selectivity of breathing MOF is expected always to be lower than that of gate‐opening MOF.

Thus, to achieve optimal separation performance, the transition pressures and hysteresis widths of the individual adsorption/desorption isotherms must be considered when specifying the separation conditions. The use of the adsorption branch only (particularly if the gate opening conditions for the second component are not fulfilled) will lead to an overestimation of the separation performance. Breathing MOFs are expected to have lower selectivity in comparison to gate‐opening MOFs due to the intermediate narrow pore phase and the absence of the highly selective closed pore phase.^[^ ^37b^ ^]^The highest selectivity for the particular MOF can be achieved if the adsorption–desorption hystereses of single components are clearly separated on the pressure/concentration axis.

Hence, in order to predict selectivity, it is essential to report isotherm adsorption and desorption data, ideally in a digital format based on IUPAC recommendations.^[^ ^98^ ^]^ Such an approach will give a wider community the opportunity to screen and evaluate potential applications if flexible MOFs in a variety of fields with AI tools and machine learning and validate the hypothesis outlined here. Moreover, it would reduce ambiguity in adsorption data using IUPACs established and well‐defined units for the adsorbed amount (mmol g^−1^) and improve reproducibility.

## 3 Advanced Characterization Techniques

### 3.1 Monitoring the Framework Transitions

In this section, we emphasize the importance of a mechanistic understanding of the guest‐induced transitions of the flexible host structure at the atomic level, which is important to understand the thermodynamics of the transition and follow the changes in porosity and pore accessibility.

In the case of “gate‐opening” MOFs, the crystal structures of the guest‐free and guest‐filled phases can be determined from ex situ measurements of solvated and degassed structures. However, such results should be interpreted with caution, as guest molecules may influence the opening degree and do not contain information about the transition pathway.^[^ ^99^ ^]^ It is, therefore, recommended to carry out in situ X‐ray diffraction measurements under conditions close to those used in the gas separation studies. In the case of minor volume changes (e.g., subnetwork displacement or linker rotation^[^ ^9^ ^]^), in situ experiments can be performed on the single crystals. Single crystal X‐ray diffraction (SCD) allows to follow the changes in the framework and, in many cases, to determine the position and occupancy of the guest molecules in the pores. Such experiments can be carried out using laboratory single‐crystal X‐ray diffractometers equipped with customized cells, adapted to specific gas loading conditions.^[^ ^100^ ^]^ In all other cases, in situ PXRD can be used.

**Figure 27:**
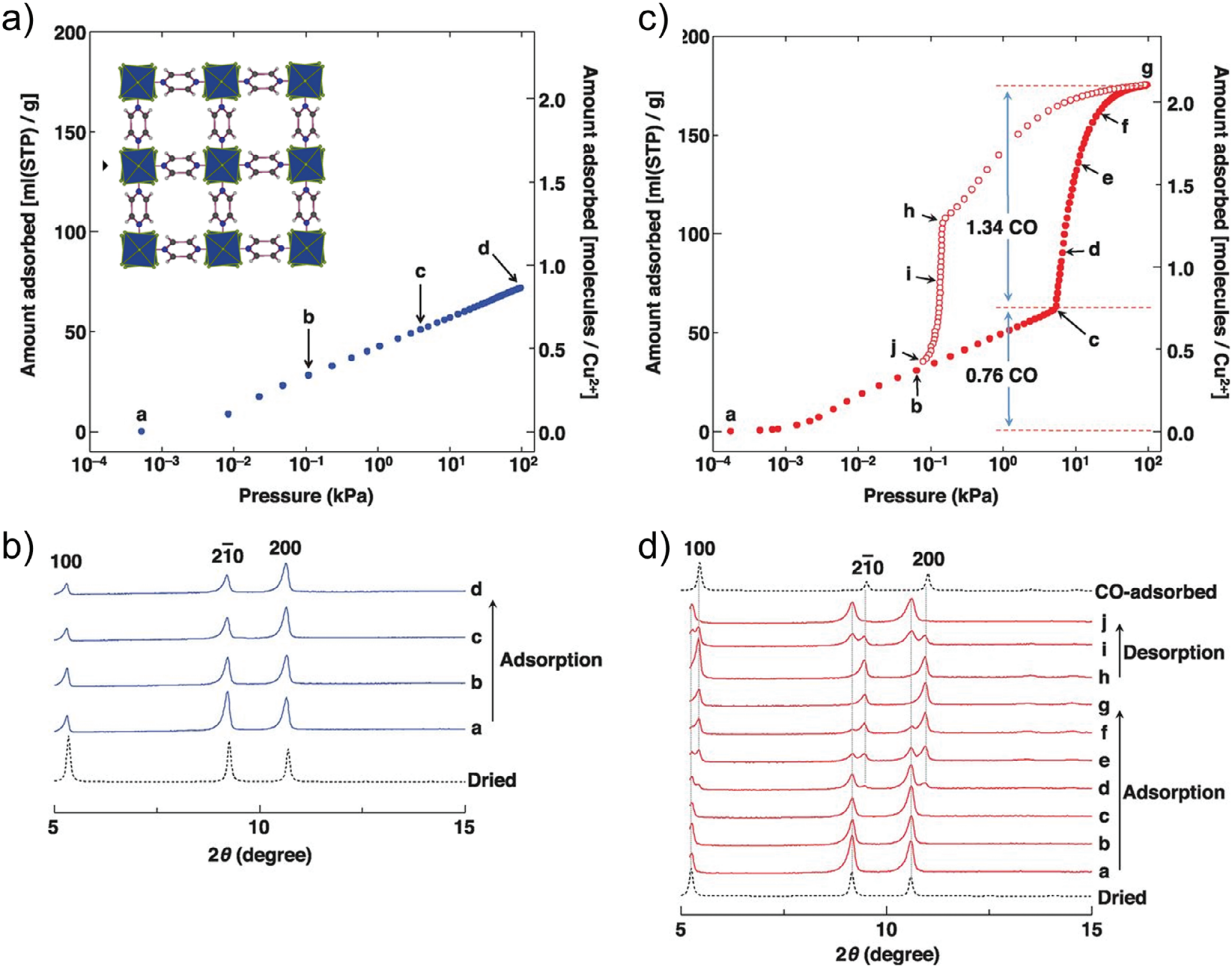
In situ PXRD/N_2_ and CO sorption measurements on [Cu(aip)]*_n_*. a) N_2_ sorption isotherms at 120 K (insert: crystal structure of NbOFFIVE‐1‐Ni). b) PXRD patterns measured at a–d points shown in the N_2_ sorption isotherms in Figure 27a. The calculated pattern for the dried [Cu(aip)]*_n_* is shown at the bottom. c) CO sorption isotherms at 120 K. Adsorption and desorption branches are shown in solid and open circles, respectively. d) PXRD patterns measured at a–j points of the CO sorption isotherm shown in Figure 27c. The c patterns for the dried [Cu(aip)]*_n_* and CO‐adsorbed phases are shown at the bottom and top, respectively. Reproduced with permission.^[^ ^101^ ^]^ Copyright 2014, American Association for the Advancement of Science.

In some cases, unexpected mechanisms behind the selectivity can be revealed, for example, self‐accelerated and selective CO sorption in [Cu(aip)(H_2_O)]*_n_* (aip – 5‐azidoisophthalate) reported by Kitagawa and co‐workers.^[^ ^101^ ^]^ In situ PXRD studied in parallel to N_2_ and CO adsorption at 120 K (**Figure** **27**) demonstrates the mechanism of stepwise CO sorption, involving the formation of a Cu^2+^─CO bond, and inducing a global structural transformation with the expansion of the squeezed paths. This expansion promotes additional CO adsorption in the center of the channel, which is the so‐called self‐accelerating gas adsorption.

Barbour and co‐workers developed and optimized a cell for in situ single crystal X‐ray diffraction under static gas loading conditions.^[^ ^100^ ^]^ SCD was used to locate the preferable adsorption sites in [NiNbOF_5_(pyrazine)_2_]*_n_* (NbOFFIVE‐1‐Ni) — one of the state‐of‐the‐art frameworks for direct CO_2_ capture from air.^[^ ^102^ ^]^ Interestingly, the CO_2_ molecule occupies an energetically favorable position, where the electropositive carbon of the CO_2_ is surrounded by four electronegative fluorine centers from four distinct (NbOF_5_)^2−^ pillars and the electronegative oxygen atoms of the CO_2_ are encaged by pyrazine hydrogens.

**Figure 28:**
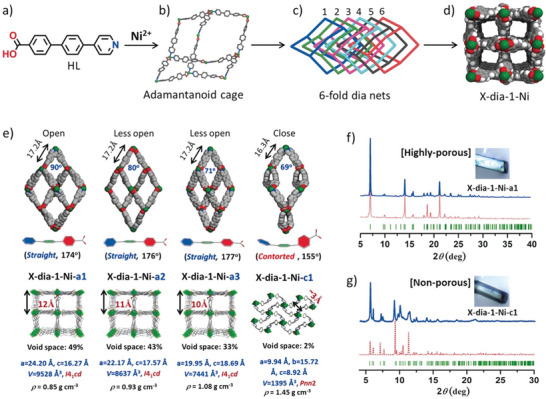
Crystal structures of NiL_2_. a) Structure of the ligand. b) Adamantanoid cage in NiL_2_. c) Sixfold interpenetrated **dia** nets in NiL_2_. d) Rectangular channels viewed along the *c*‐axis. e) Single crystal structures of the porous (**a1**–**a3**) and nonporous (**c1**) phases of X‐dia‐1‐Ni. f) Crystal morphology and PXRD pattern of **a1**. g) Crystal morphology and synchrotron PXRD (*λ* = 0.8262 Å) pattern of **c1**. Reproduced with permission.^[^ ^39^ ^]^ Copyright 2018, Wiley‐VCH GmbH & Co. KGaA.

The cascade of the phase transitions was observed during the desolvation of the adamantoid framework with a composition [NiL_2_]*_n_* (L = 4‐(4‐pyridyl)‐biphenyl‐4‐carboxylate).^[^ ^39^ ^]^ All structures were solved and refined from the single crystal X‐ray diffraction data collected on the single crystal with CH_2_Cl_2_ in the pores at different stages of degassing. As a result, three different structures, **a1**, **a2**, and **a3**, all crystallizing in the same *I*4_1_cd space group, were visualized. Desolvation of the structure leads to reductions in cell volume (9528, 8637, and 7441 Å^3^ for **a1–a3**, respectively) and solvent‐accessible void volume (49%, 43%, and 33% for **a1**–**a3**, respectively) induced by the changes in N–Ni–N/C–Ni–C bond angles: 93.0°/101.5°, 90.7°/101.4°, and 88.0°/102.4° for **a1**–**a3**, respectively (**Figure** **28**). The heating of **a1–a3** phases in vacuum yields the completely desolvated and dense X‐dia‐1‐Ni‐c1 structure, showing only 2% of the solvent‐accessible void. In the same work, the authors followed the CO_2_ physisorption by PXRD, indicating the reversibility of the transitions. Moreover, multistep isotherm was observed in high‐pressure methane physisorption, which served as a valid precondition for the gas separation studies.

**Figure 29:**
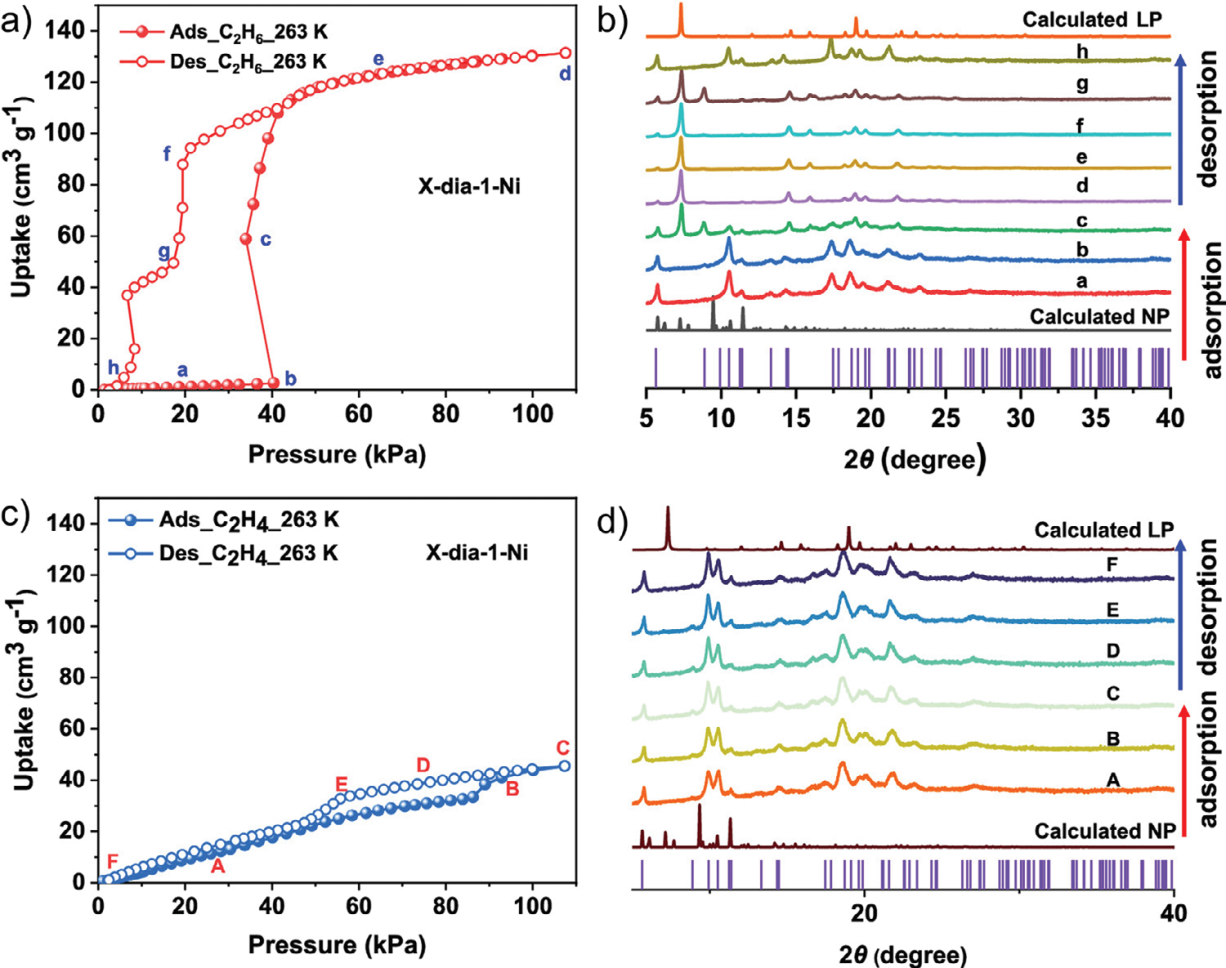
a) C_2_H_6_ physisorption isotherm at 263 K. b) Corresponding PXRD patterns collected in situ. X‐dia‐1‐Ni undergoes a reversible transformation from an np phase to an lp phase upon ethane dosing. c) C_2_H_4_ physisorption isotherm at 263 K. d) Corresponding in situ PXRD patterns. In situ PXRD patterns indicate that the desolvated phase adsorbs C_2_H_4_, but C_2_H_6_ is not adsorbed until phase transformation occurs. Reproduced with permission.^[^ ^103^ ^]^ Copyright 2024, American Chemical Society.

In the follow‐up manuscripts, authors developed the strategy for controlling “gate opening” pressure by utilizing a multivariate approach and mixing Ni and Co in the structure.^[^ ^40^ ^]^ Here, in situ PXRD were collected upon high‐pressure methane physisorption, indicating the phase transition of the framework. Finally, the mixed‐metal MOF with a composition X‐dia‐1‐Ni_0.89_Co_0.11_ was tested in C_2_H_6_/C_2_H_4_ separation and indicated high inverse selectivity toward C_2_H_6_ with 9.1 times higher uptake compared to C_2_H_4_. In situ PXRD experiments were conducted on X‐dia‐1‐Ni at 263 K, indicating the phase transition at 40 kPa upon C_2_H_6_ adsorption, whereas no changes were observed upon the physisorption of C_2_H_4_ in the same conditions (**Figure** **29**).

**Figure 30:**
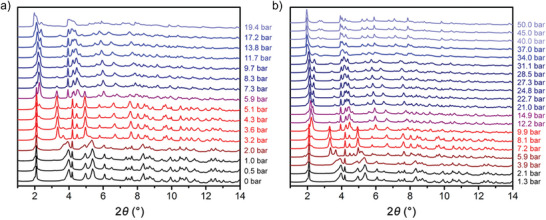
Powder X‐ray diffraction data for [Co(bdp)]*_n_* dosed with a) pure CO_2_ and b) a 50:50 mixture of CO_2_/CH_4_ over a range of pressures. In both data sets, the abrupt appearance or disappearance of peaks indicates discrete phase changes, whereas gradually shifting peaks indicate gradual framework expansion/contraction. Reproduced with permission.^[^ ^69^ ^]^ Copyright 2018, American Chemical Society.

As discussed above, [Co(bdp)]*_n_* compound is a promising candidate in terms of gas storage and separation. The structural flexibility was investigated by in situ PXRD in parallel to high‐pressure physisorption of pure CO_2_ and CO_2_/CH_4_ mixture at 298 K (**Figure** **30**).

**Figure 31:**
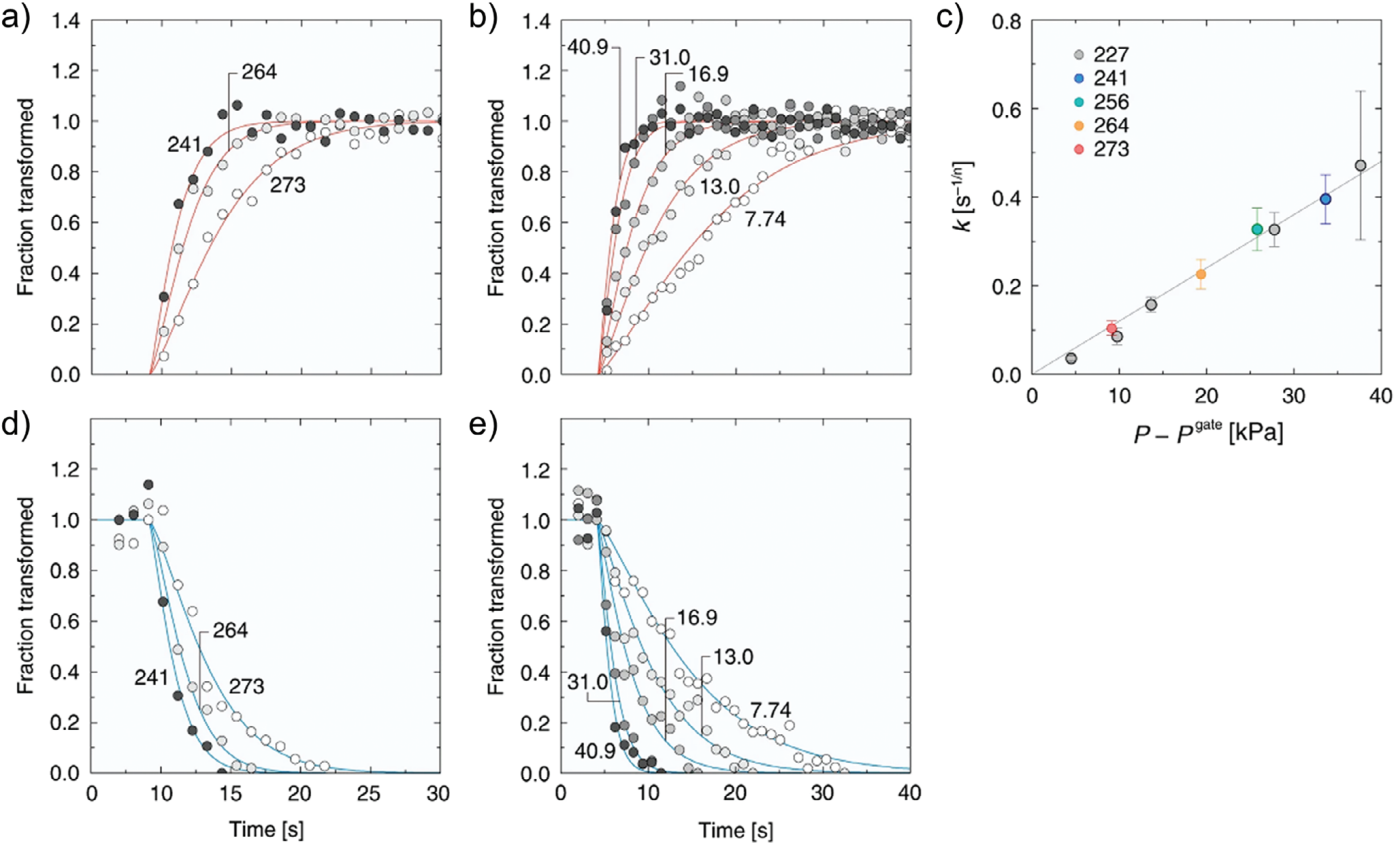
In situ time‐resolved PXRD/CO_2_ physisorption on ELM‐11: a) fractions of the open phase and d) for the closed phase at 40.8  kPa and 241 K, at 41.0 kPa and 264 K, and at 40.8 kPa and 273 K. The numbers in (a) and (d) denote the temperature. b) Fractions of the open phase, and e) for the closed phase at 227 K as a function of the CO_2_ pressure. The numbers in (b) and (e) denote the CO_2_ gas pressure in kPa. The curves after 4.15 s in (a–e) were obtained by fitting the KJMA equation. c) Relationship between the rate coefficients and the pressure difference between the CO_2_ gas pressure, *p*, and the gate‐opening pressure, *p*_go_. The error bar represents the standard deviation of the value obtained using the least‐square fitting of the KJMA equation to the experimental data (*n* ≥ 3, *n*: number of experimental points used for fitting). Reproduced with permission.^[^ ^22^ ^]^ Copyright 2020, The Authors, under CC BY 4.0.

Tanaka and co‐workers studied the rearrangement of ELM‐11 framework upon CO_2_ adsorption by time‐resolved in situ PXRD at different temperatures and threshold pressures to evaluate the switching kinetics in the powdered sample (**Figure** **31**).^[^ ^22^ ^]^ The authors conducted experiments at different incrementally increasing CO_2_ pressures all above *p*_go_, showing dependence between the rate constant and the *p*–*p*_go_ pressure difference, where *p* is the CO_2_ pressure introduced.

To study hydrogen isotope separation, neutron powder diffraction became a useful technique because of the high elastic scattering length of deuterium. In such a case, both the information about the host and guests can be extracted. Oh and co‐authors demonstrated that the physisorption of H_2_ and D_2_ in MIL‐53(Al) at cryogenic temperatures proceeds in different ways.^[^ ^94^ ^]^ While hydrogen shows a one‐step isotherm, two steps are observed in deuterium isotherm, measured at the boiling point. Neutron powder diffraction (NPD), conducted at defined gas loadings, indicates contraction in the case of hydrogen. The same experiment with deuterium indicates the contraction and reopening of the structure and shines a light on the mechanism of selective isotope adsorption. Due to nuclear quantum effects, deuterium shows higher enthalpy of adsorption, which is sufficient to open the structure and keep it in an open pore state. Besides NPD experiments, proving the structural changes, authors conducted in situ quasi elastic neutron scattering experiments, which showed that the mobility of the hydrogen in this clamped mode is less than that when the molecule is in the open structure.

In summary, the in situ scattering techniques provide unique structural information on the dynamics of the host framework. The obtained structures can be used not only for the evaluation of geometrical porosity but also for more complex calculations, such as GCMC, osmotic energy, and molecular dynamics simulations. In some particular cases, information about the framework dynamics and guests can be gained. However, much more information on the guest's behavior upon adsorption can be derived from spectroscopic techniques, in particular, one that can differentiate between adsorptive and adsorbate.

### 3.2 Monitoring the Guest Behavior

The monitoring of the guest molecules u the adsorption process in situ is crucial for understanding the role of the guests for the flexibility and adsorption process.

The main drawback of volumetric coadsorption techniques, usually used to measure mixed gas adsorption isotherms, is the limited temperature and pressure range, which is indeed quite close to the real‐world application for CO_2_/N_2_, CO_2_/CH_4_, and alkane/alkene separation. However, it technically does not cover the cryogenic temperatures and low‐pressure range typical for hydrogen isotope separation and has certain limitations in terms of the gas mixture composition. In order to analyze the selectivity at cryogenic temperatures, TDS can be used, where the gas mixture is adsorbed first, followed by applying an ultrahigh vacuum to remove unadsorbed gas. The desorbed gases are then monitored by mass‐spectroscopy upon heating, which allows for the estimation of the interaction strength and quantification of the species desorbed.^[^ ^94^, ^97^, ^104^ ^]^

In situ NMR is an advanced technique that differentiates and quantifies the fluid in the gas and adsorbed phases, assuming that all gases contain an NMR active nucleus. NMR offers the advantage of not only enabling quantification but also providing insights into the mobility of molecules.

Brunner and co‐workers established a high‐pressure in situ cell connected to the homemade gas mixing unit.^[^ ^97^ ^]^ The instrumentation was used to evaluate the coadsorption of CO_2_/CH_4_ mixture in DUT‐8(Ni), SNU‐9, and JUK‐8 (as discussed in Section 2.2.1).^[^ ^43^, ^86^ ^]^ The same setup was used for the analysis of krypton/xenon coadsorption on DUT‐8(Ni) at 280 K. ^129^Xe NMR experiments on rigid and flexible DUT‐8(Ni) at 283 and 237 K using a mixture of xenon and krypton indicates a clear decrease in the chemical shift pointing on the partial replacement of xenon by krypton, i.e., krypton coadsorption. Analysis of the spectra suggests a slightly higher selectivity for xenon adsorbed in the flexible version of DUT‐8(Ni). This can be explained by the overlapping the hysteresis in Xe in Kr isotherms in the analyzed pressure range; namely, once the “gate opening” pressure for xenon is reached and the framework is open, coadsorption of krypton will not change the thermodynamics, op phase remains energetically favorable phase.

### 3.3 Combination of Different Techniques

**Figure 32:**
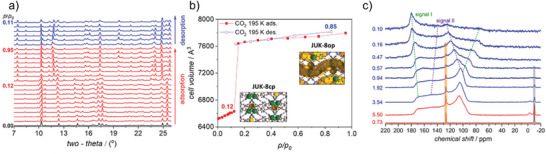
Evaluation of JUK‐8 behavior in CO_2_/CH_4_ adsorption: a) PXRD patterns collected upon CO_2_ physisorption. b) Evolution of unit cell volume upon CO_2_ physisorption, and c) in situ ^13^C NMR in parallel to CO_2_/CH_4_ (50:50) physisorption at 195 K. Reproduced with permission.^[^ ^86^ ^]^ Copyright 2021, The Authors. Published by American Chemical Society. This publication is licensed under CC‐BY 4.0.

The most efficient way to study the gas separation in flexible MOF is to apply multiple in situ techniques, as shown by Roztocki et al. for JUK‐8 in the mixed gas CO_2_/CH_4_ adsorption (**Figure** **32**).^[^ ^86^ ^]^ In order to explain the observed performance, in situ PXRD was conducted at 195 K, indicating the phase transition from the JUK‐8_cp to the JUK‐8_op phase. To characterize the selectivity in the low‐temperature regime, in situ ^13^C NMR coadsorption experiment using a ^13^CO_2_/^13^CH_4_ mixture (molar ratio 1:1) at 195 K was conducted. The analysis of the spectra indicates that only minor amount of methane coadsorbs on JUK‐8op@CO_2,_ even at low temperatures and high pressure. In situ IR spectroscopy conducted under CO_2_ loading suggests the changes in the host structure, namely, changes in the O–C–O asymmetric stretching region of the oba^2−^ carboxylate linkers. In addition, two signals from the adsorbed CO_2_ (at 2340 and 2376 cm^−1^) were observed in contrast to one signal observed for gaseous CO_2_ (2345 cm^−1^).

In summary, the application of in situ diffraction techniques is crucial for understanding the behavior of the host structure and guest molecules upon the physisorption of the gas mixtures. The results provide important information about the host structure under defined conditions, which is essential for understanding the material performance and may be used as input for in silico studies. Spectroscopic techniques, applied at desired gas pressure/temperature conditions, can clearly differentiate between adsorbed and uadsorbed guest molecules and provide direct proof of the separation performance.^[^ ^55^, ^97^, ^105^ ^]^

## 4 Challenges to Solve for the Application of Flexible Adsorbents

### 4.1 Kinetics of Switching

When considering flexible MOFs as adsorbents, not only the working capacity and selectivity play a crucial role. The kinetics of switching between the phases have to be taken into account as well. The adsorption kinetics, in this case, is not only dependent on the crystal surface barriers and gas diffusion kinetics but arises from the framework bistability and is coupled to the physical properties of the guest molecules with characteristic barriers of fluid nucleation, diffusion, and adsorption processes.^[^ ^106^ ^]^ Although the importance of kinetics has been known for over 15 years,^[^ ^107^ ^]^ the kinetics of the adsorption process in flexible MOFs is still underexplored.^[^ ^108^ ^]^

The most frequently applied method to analyze the kinetics of gas‐induced phase transition in an ensemble of crystals (powder or pellets) is to monitor the pressure drop in the adsorption cell. This method is also applied in the industry to evaluate adsorbents, particularly for pressure swing adsorption, with high cycling rates. However, this method does not analyze the intrinsic switching but the macroscopic gas uptake of the sample, which also involves mass transport and is therefore highly dependent on the reactor volume and sample amount.

**Figure 33:**
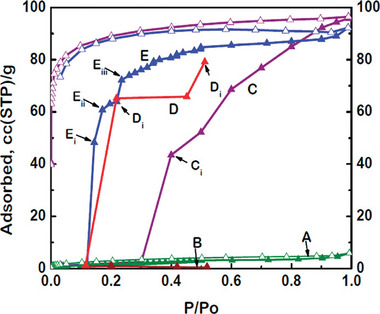
Nitrogen adsorption on RPM3_Zn at 77 K shows a pronounced effect of (fictives) exposure time (d*t*): (A) d*t* = 5 s, (B) d*t* = 20 s, and (D, E) d*t* = 360 s collected by ASAP 2020 from Micromeritics. (C) Collected using a fresh sample and Autosorb‐1 MP from Quantachrome Instruments. The allowed time varied from 6 min to 3.7 h for adsorption. A history effect is also observed in which the number of data points affects the uptake (e.g., screening experiment D with 4 points versus full isotherm E with 45 points). Times (from the previous data point) for select points are Di = 67 h, Dii = 8.5 h, Ei = 62 h, Eii = 20 h, and Eiii = 14 h. The total times for adsorption (in hours) are A ≈ B (∼1) < C (16) < D (80) < E (135). Prior to the sharp rise, curves C–E follow the A and B curves. Reproduced with permission.^[^ ^109^ ^]^ Copyright 2011, American Chemical Society.

In such a way, Li and co‐workers investigated the rate of adsorption coupled with the structural transition in a flexible RPM3_Zn ([Zn_2_(bpdc)_2_(bpee)]*_n_*, bpdc = 4,4′‐biphenyldicarboxylate; bpee = 1,2‐bipyridylethene).^[^ ^109^ ^]^ The compound exhibits a significant induction period for opening by N_2_ and Ar at low temperatures (**Figure** **33**). Such a long induction period is not observed for H_2_ or O_2_ at comparable pressures and temperatures, suggesting the rate of opening is strongly influenced by the gas–surface interaction rather than external stress.

The induction period leads to severe mass transfer limitations for adsorption and overestimation of the gate‐opening pressure. The authors reviewed several adsorption rate models and found that none adequately describes the experimental rate. Statistically, the rate data are best described by a compressed exponential function. The resulting fitted parameters exceed the expectations for adsorption but fall within those expected for phase transition.^[^ ^109^ ^]^

By treating adsorption as a phase transition, the generalized Avrami theory of phase transition kinetics was used to describe adsorption in flexible hosts.

Gläser and co‐workers^[^ ^110^ ^]^ investigated the kinetics of the *n*‐butane and *iso*‐butane gas mixture adsorption on gate pressure Cu‐IHMe‐pw ([Cu_2_(H‐Me‐trz‐Ia)_2_])*_n_* ^[^ ^111^ ^]^ MOF. The uptake curves reveal complex interactions on the outer surface of MOF particles. The overall rate of adsorption‐induced structural transition depends on several factors, including degree of pressure rise, temperature, particles size, and the subsequent diffusion rate into newly opened pores. With the aid of a kinetic model based on the linear driving force (LDF) approach, both rates of diffusion and structural transition were studied independently of each other. The authors claim that the overall velocity of gas uptakes in flexible MOFs is predominantly determined by the rate of the structural transition.

**Figure 34:**
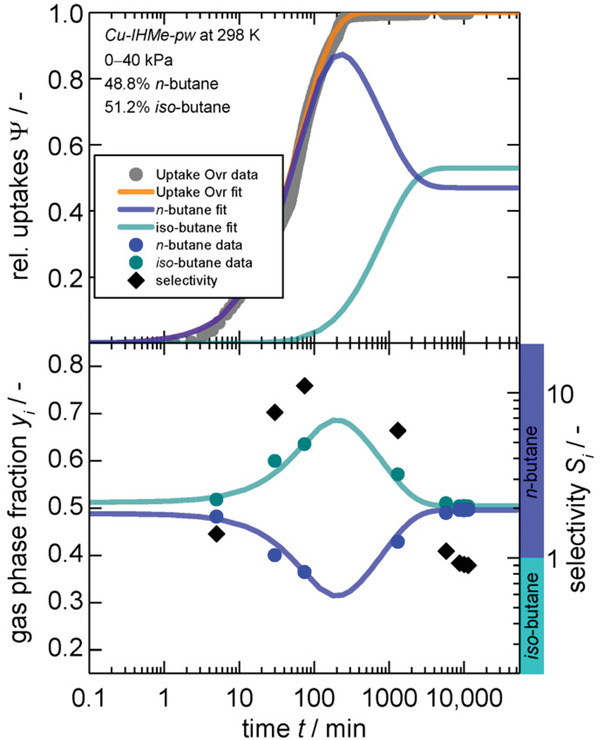
Kinetic of gas uptake (top), gas phase composition (bottom, left axis), and adsorption selectivity (bottom, right axis) in dependence of time for an equimolar mixture of *n*‐butane and *iso*‐butane on Cu‐IHMe‐pw for a pressure jump of 0–40 kPa. Additionally, the overall gas phase compositions and sorption kinetics are modeled by the “GO” model and a mass balance. Within the bottom graph, a selectivity larger than 1 indicates a preference for *n*‐butane, as indicated by the additional colored ribbons. It should be noted that a 50:50 molar mixture was aimed for, but actual results show slight deviations with 48.8 to 51.2. Reproduced with permission.^[^ ^110^ ^]^ Copyright 2024, The authors. Licensee MDPI, Basel, Switzerland. Distributed under the terms and conditions of the CC BY 4.0.

It could also be shown that the overall gas uptake is slower for the gas mixture as compared to the single‐gas uptake of *n*‐butane with the same partial pressure step, although much faster than compared to the bare *iso*‐butane adsorption (half coverage is reached after 40 min for the mixture, 1 min for *n*‐butane and ≈1000 min for *iso*‐butane individually) (**Figure** **34**). From the evolution of the gas phase composition, it could be seen that *n*‐butane is predominantly adsorbed at the beginning, reducing the total gas‐phase fraction to ≈33%, resulting in a total separation factor of a maximum of 10 (at time 200 min). Beyond that, *iso*‐butane continuously enters the opened framework, exchanges the adsorbed *n*‐butane and incorporates itself within the framework. This leads to a subsequent increase in the gas phase fraction of *n*‐butane and a final separation factor of 0.9 after 10 000 min (≈7 days) for *n*‐butane.

In recent years, new methods have been proposed to investigate the kinetic of phase transitions, involving in situ (single crystal) XRD, optical, scanning electron microscopy (SEM), and atomic force microscopy (AFM).

The phase transformation of a [Zn_2_(1,4‐ndc)_2_(dabco)]*_n_* (1,4‐bdc = 1,4‐benzenedicarboxylate) crystal was investigated in situ by AFM (with a time resolution of 40 s in DMF solutions with various concentrations of the biphenyl as a guest molecule) by the group of Kitagawa.^[^ ^112^ ^]^ It was found that the lattice structure of the liquid–solid interface quickly changed (within ≈10 min) in response to the biphenyl concentration change. It should be pointed out that in many cases, it is not only the analytical instrument limiting the temporal resolution but also the realization of a stepwise stimulus increase that poses challenges because of mass transfer limitations and diffusional broadening of a pressure or concentration pulse peak.

Phase changes were monitored on ≈1 µm single crystals of MIL‐53(Al) by in situ environmental SEM.^[^ ^113^ ^]^

**Figure 35:**
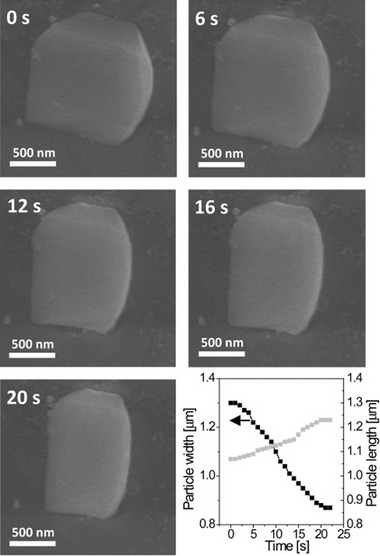
SEM images of MIL‐53(Al) obtained at 0, 6, 12, 16, and 20 s of beam irradiation; bottom right, particle width and length as a function of observation time. Reproduced with permission.^[^ ^113^ ^]^ Copyright 2014, Elsevier Inc.

The deformation of single crystals was observed over several seconds upon electron beam irradiation, which caused partial desorption of the toluene from the pores. The phase transitions were assigned through the detected variations in crystal shape (**Figure** **35**), which was further supported by TEM measurements.

**Figure 36:**
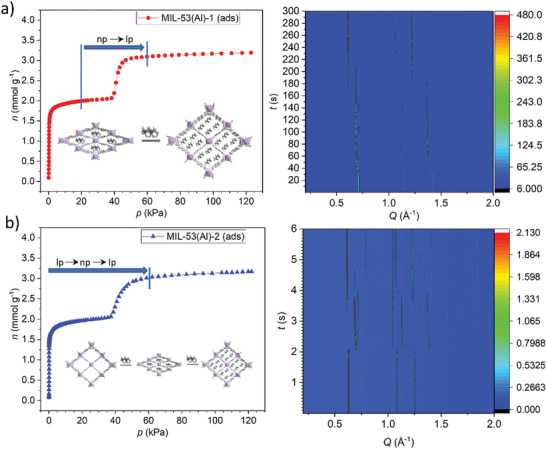
In situ time‐resolved PXRD measured during the adsorption of *n*‐butane at 298 K on MIL‐53(Al): a) reopening of the crystals with average crystal size of 28 ± 18 µm, and b) breathing transition in the crystals with average crystal size of 1.2 ± 0.5 µm. Reproduced with permission.^[^ ^114^ ^]^ Copyright 2022, Royal Society of Chemistry.

Bon and co‐workers demonstrated that the phase transition kinetic in the MIL‐53(Al) system is significantly controlled by the particle size (**Figure** **36**).^[^ ^114^ ^]^ Samples with the average particle size of 1.2 and 28 µm were compared upon adsorption of *n‐*butane at 298 K. It was also found that the contraction transition (op–np) is the fastest transition for both particle size regimes and the reopening of the structure proceeds much slower. Hence, the activation barrier for opening the narrow, butane‐filled pore is larger, probably due to the diffusion limitations of the guest.

**Figure 37:**
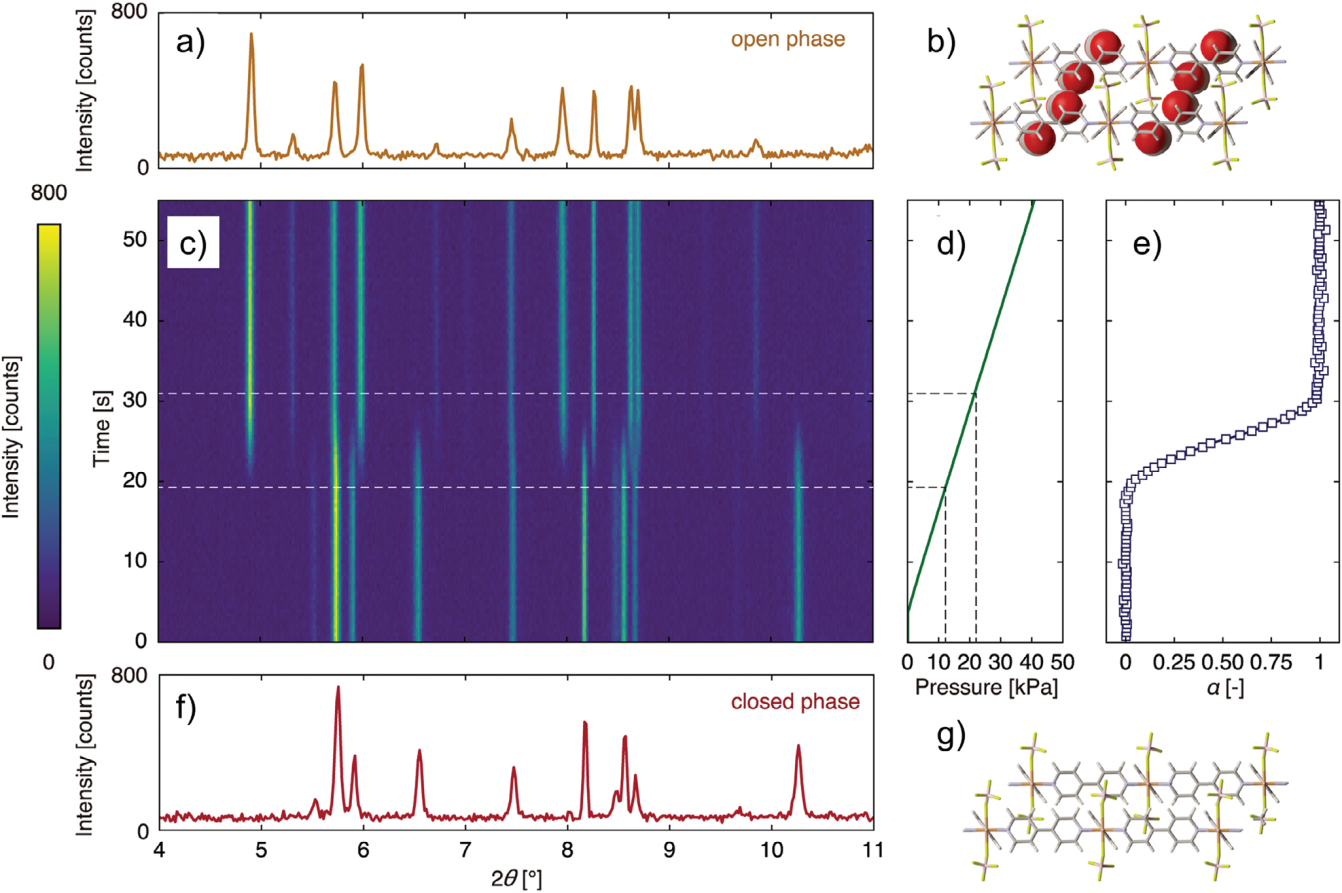
a) XRD pattern at 42 kPa CO_2_ and b) crystal structure of ELM‐11 in the open pore phase. c) Colormap of time resolved XRD patterns of ELM‐11 at 248 K. CO_2_ was introduced 5 s after the start of the measurement with the constant flow rate of 0.8 kPa s^−1^, until   42 kPa pressure was reached. e) Time evolution of fraction transformed, calculated from the peak intensity ratio from the XRD patterns in (a) and (f) at regular intervals of time. f) XRD pattern at 0 kPa and g) crystal structure of ELM‐11 in the closed pore phase. Reproduced with permission.^[^ ^115^ ^]^ Copyright 2023, The Authors, under CC BY 4.0.

The CO_2_‐induced gate opening of ELM‐11 was investigated by time‐resolved in situ PXRD (**Figures** 31 and **37**), and a theoretical kinetic model of this process was developed to gain atomistic insight into the transition dynamics.^[^ ^115^ ^]^

The developed model consists of the differential pressure from the gate opening (indicating the ease of structural transition) and reaction model terms (indicating the transition propagation within the crystal).

**Figure 38:**
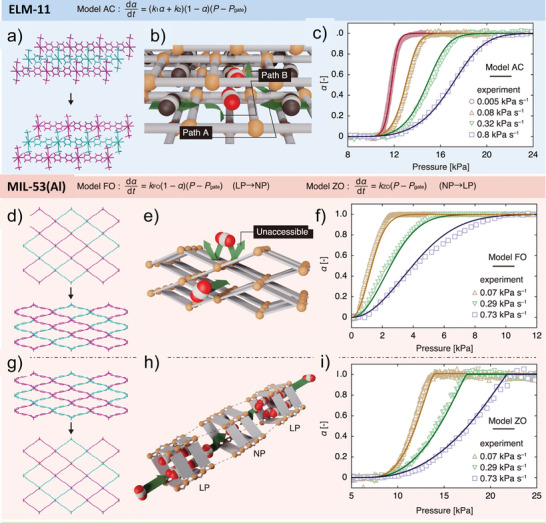
Atomic structure, a schematic of the structural transition, and measurement and calculation results for a–c) the gate opening of ELM‐11 at 248 K, d–f) op → np transition of MIL‐53(Al) at 223 K, and g–i) np → lp transition of MIL‐53(Al) (at 195 K). Reproduced with permission.^[^ ^115^ ^]^ Copyright 2023, The Authors, under CC BY 4.0.

The reaction model of ELM‐11 is an autocatalytic reaction with two pathways for CO_2_ penetration into the framework. However, gas adsorption analyses of MIL‐53(Al), possessing different mechanisms of phase transitions (breathing), indicate that the kinetics of the adsorption‐induced structural transition is highly dependent on framework structure (**Figure** **38**). The authors confirm the differences in the kinetics of op to np and np to op transitions, consistent with the work of Bon et al.^[^ ^114^ ^]^

The rate of structural transition of gating in ELM‐11 investigated by time‐resolved in situ synchrotron Pin orderXRD measurements shows that the structural transition started immediately after the introduction of CO_2_ at 40.8 kPa and 273 K and was accomplished in ≈10 s.^[^ ^22^ ^]^ The MOF accommodating CO_2_ responded quickly to the decrease in gas pressure at 273 K: the structural transition was completed in 5 s when the CO_2_ pressure was decreased at the rate of 2.4 kPa s^−1^. Moreover, the rate of phase transition increases as the temperature decreases under the same CO_2_ pressure. At 227 K the rate of structural transition increased with increasing CO_2_ pressure, and the phase transition was completed within a few seconds at the highest gas pressure. Furthermore, these data were found to obey the Kolmogorov–Johnson–Mehl–Avrami (KJMA) equation.^[^ ^22^ ^]^

Kaskel and co‐workers investigated the phase transition kinetics of DUT‐8(Ni) using three different techniques.^[^ ^106^ ^]^ The newly designed microfluidic breakthrough apparatus allowed observation of transformation times for individual crystals varying with respect to Ni/Co ratios. Comparison with the in situ PXRD data led to the conclusion that the macroscopically observed switching rate is mainly governed by variations in the induction period. The transformation of individual crystals is much faster than that of an ensemble of crystals, which is, therefore, not easily observable. Crystals with a higher activation barrier (corresponding to a higher gate opening pressure) show a longer induction period, leading to an overall slower adsorption kinetic of the ensemble. However, the individual crystal transformation rate is below 1 s, which was the temporal limit of resolution in this study. The exact and intrinsic individual crystal transformation rate remains uncovered, and novel methods approaching a temporal resolution in the ms or s regime are still to be developed in order to shed light on this aspect.

### 4.2 Volume Change of the Crystals Associated with Phase Transition

Although network flexibility gives rise to significant advantages for gas storage and separation applications, it also poses many challenges. The structural phase transitions are typically associated with macroscopic changes in crystal size.^[^ ^116^ ^]^ This aspect has been beneficially used for actuators and sensors.^[^ ^117^ ^]^

ELM−11 shows two steps upon the CO_2_ adsorption, with a 28% expansion in the interlayer distance in the first transition and a 56% expanded layer structure compared to the initial structure in the second one.^[^ ^118^ ^]^ The macroscopic changes in the powder bed upon CO_2_ adsorption have been reported already in 2006.^[^ ^119^ ^]^

Kaskel and co‐workers studied volume expansion and force exerted by flexible MOFs through expansion for MIL‐53(Al).^[^ ^117b^ ^]^ The effect of the packing density on the mechanical response was also evaluated. Three different regimes were identified according to the packing density. The pressure gained from the opening step was found to be higher than that needed to compress the empty framework and is specific for the stimulating fluid.

The macroscopic expansion, however, creates challenges in adsorptive storage and separation applications, especially in the adsorption bed. In most applications, the adsorption column is several meters high, and thus, the adsorbent at the bottom of the bed experiences significant gravitational force due to the high weight of the packed bed above. This lower part of the bed has to exert a significant force against the upper part to open its pores and accomplish the volume change. In extreme cases, pore expansion may be completely suppressed in such a real macroscopic application. Another aspect is that the adsorber column has to provide enough dead volume for expansion, which may limit volumetric efficiency, which is often overlooked in academic research. Moreover, the expanding adsorbent will most likely induce a pressure drop in the column.^[^ ^46a^ ^]^ Hence, MOFs showing minimal volume changes or flexibility based on the internal movement of the building blocks are more favorable.

### 4.3 Crystal Damage Associated with Phase Transition

The huge volume change associated with the mechanical and thermal stress often leads to the damage of the crystals (adsorption‐induced milling).^[^ ^120^ ^]^ The magnitude of the effect depends on the structure and chemical composition of the MOF itself, as well as on the particle size, adsorptive, and adsorption temperature.

MIL‐53(Al) and ELM‐11 were proven to be stable in cyclic experiments with butane at 298 K, withstanding 100 adsorption/desorption cycles without significant changes in performance.^[^ ^120^ ^]^ Also repeated pressure swings between 0.5 and 2.0 MPa at 298 and 323 K upon six CO_2_ adsorption/desorption cycles for MIL‐53(Al) did not cause the deterioration in the adsorption capacity.^[^ ^121^ ^]^ For ELM‐11, the reproducibility of the gating was demonstrated in 50 cycles of CH_4_ physisorption at 303 K.^[^ ^34^ ^]^

In contrast, for DUT‐8(Ni) and SNU‐9, the multiple adsorption/desorption stress upon butane adsorption at room temperature leads to the reduction of crystallite size, causing changes in the switching behavior in the initial 10 physisorption runs, and a characteristic shift of the “gate‐opening” pressure to higher values is observed.^[^ ^120^ ^]^ The reason for such behavior is particle size‐dependence t flexibility.^[^ ^48^ ^]^

The comprehensive investigations show the existence of three main particle size regimes in the case of DUT‐8(Ni): Several micrometer large particles show pronounced gating behavior, while ≈500 nm–1 µm sized particles reveal a suppressed opening (stabilization of the metastable cp phase). Particles with sizes below 500 nm have suppressed closing ability (stabilization of the op phase).^[^ ^48^ ^]^

Abylgazina et al.^[^ ^48b^ ^]^ demonstrated that the changes in the gate opening pressure with particle size are not linear and follow a logarithmic dependence on the facet extension. Moreover, the steepness of the adsorption branch in the gating region decreases with decreasing crystal size, pointing to the significantly broader activation energy distribution in the small grains compared to the large crystallites. The threshold of the particle size, at which the compound experiences rigidification and loss of crystallinity, depends in the DUT‐8(M) (M = metal) system, on the metal type in the paddle wheel.^[^ ^48^, ^122^ ^]^ The effects of crystal size and morphology are critically discussed in a recent review.^[^ ^48^ ^]^

### 4.4 Change in the Flexibility Characteristics due to the Additives and Shaping

When building an adsorber for gas separations, the adsorbing material is usually pelletized to improve the gas flow, reduce pressure drop, and improve overall performance. For MOF materials, this poses a significant challenge since many of the well‐known frameworks cannot withstand the stress of shaping into pellets and, as a consequence, important properties, like sorption capacity and selectivity, are reduced.^[^ ^82^, ^123^ ^]^ In flexible MOFs, loss or massive impact on the flexibility characteristics can be expected in addition to mechanical deterioration.^[^ ^124^ ^]^

**Figure 39:**
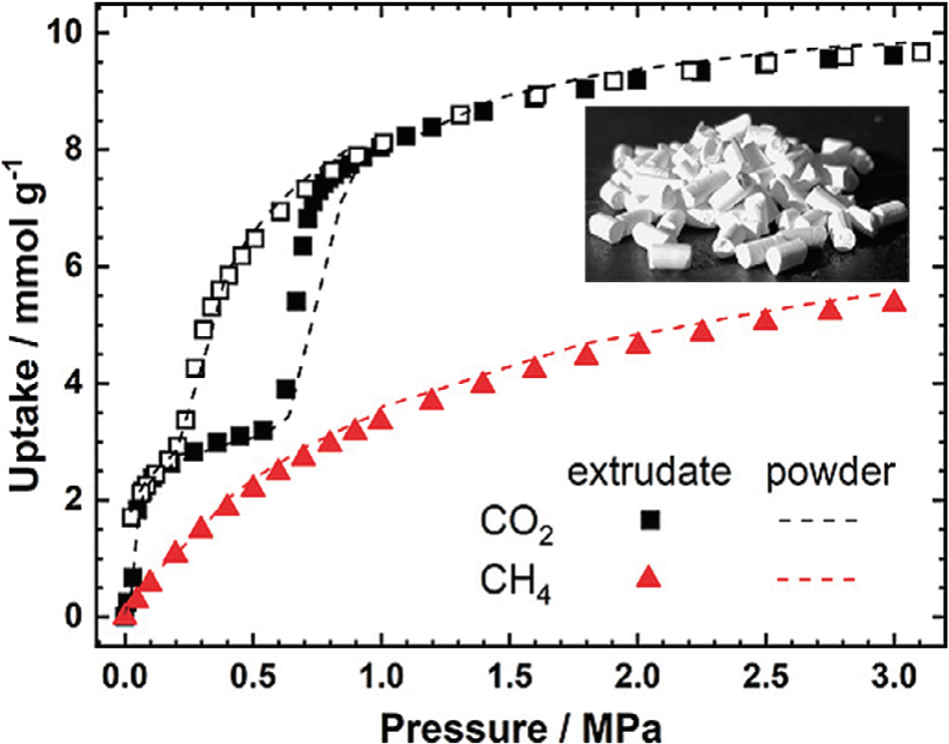
High‐pressure methane (red) and carbon dioxide (black) physisorption isotherms at 303 K on MIL‐53 with 3 wt% of methyl cellulose as binder (dashed lines) in comparison to the adsorption isotherms on MIL‐53 powder (symbols). Reproduced with permission.^[^ ^125^ ^]^ Copyright 2019, Wiley‐VCH GmbH & Co. KGaA.

MIL‐53(Al), however, shows stable performance after shaping while maintaining the textural properties and characteristic framework dynamics.^[^ ^125^ ^]^ It was demonstrated that pellets with 5 or 10 wt% of binder reached mechanical resistances comparable with some of the carbon molecular sieves pellets. All experimental insights revealed that the reversible breathing effect—characteristic for MIL‐53—was preserved even though the samples were shaped into cylindrical extrudates. In situ X‐ray diffraction studies under humid conditions confirmed the similarity in phase transition kinetics for extrudates and powders. Moreover, the phase transition of the MIL‐53 extrudates upon adsorption of carbon dioxide was confirmed by high‐pressure adsorption isotherms of CH_4_ and CO_2_ on MIL‐53 powders and extrudates, which exhibited similar capacities for both gases (**Figure** **39**).

Mechanical stress can potentially induce phase transition in flexible MOFs.^[^ ^126^ ^]^ In a recent study the group of Song shows the influence of mechanical pressures on MIL‐53(Al). High mechanical pressures and temperatures support the interactions between CO_2_, and the hydroxyl groups and the Al centers of the network and increase the sorption capacity because of chemisorptive behavior.^[^ ^127^ ^]^

Kundu et al. tested the pelletization of MIL‐53(Al)‐OH and MIL‐53(Al).^[^ ^46a^ ^]^ Although no decrease in the uptake or changes in the isotherms’ shape were observed, both samples could not withstand the stress of deformation, resulting in the breaking of the pellets during CH_4_ adsorption. Similar results were shown by the group of Denayer,^[^ ^82^ ^]^ who also investigated the pelletization of MIL‐53(Al).

The application of binders to create flexible MOFs containing composites can also potentially lead to changes in the flexible behavior.

Along these lines, the Watanabe group was able to show the influence of polymer matrix (polyvinylpyrrolidone, PVP) on the switching behavior of ELM‐11.^[^ ^124^ ^]^ Later on, similar experiments were also conducted with an interpenetrated [Cu_2_(bdc)_2_(bpy)]*_n_* framework (referred to as JG‐MOF).^[^ ^128^ ^]^ Interestingly, the material shows a very distinct change in the gate‐opening behavior, depending not only on the amount of PVP added but also on the sample preparation approach. Increasing binder amount causes a “slacking” of the gate opening. The term describes an external force‐induced shift from a stepwise to a sequential transition, leading to a smearing of the originally steep adsorption branch originating from the phase transition.^[^ ^124^ ^]^ This effect was explained through free energy analysis, showing a nonsynchronized transformation within a single MOF particle. Furthermore, the slacking could be modulated by the composite preparation method as it impacts the force inflicted on the material. For higher external forces, more pronounced slacking was observed. In line with theoretical calculations, similar experiments on the less‐expanding interpenetrated JG‐MOF confirmed this theory. Due to the reduced expansion of the networks, less force was exerted in the pellet, thus minimizing slacking.^[^ ^128^ ^]^ Therefore, developing methods to reduce the stress inflicted by binders is crucial.^[^ ^124^ ^]^

### 4.5 Slipping Off Effect

**Figure 40:**
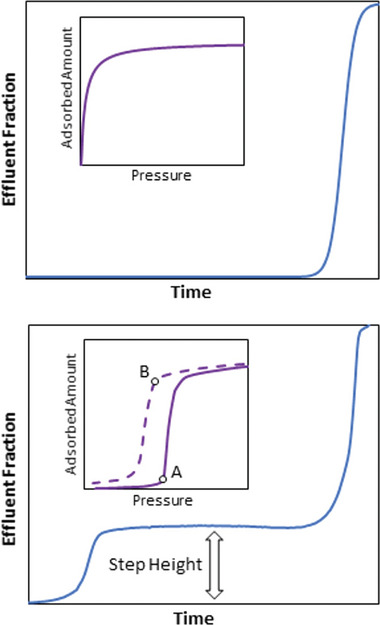
Comparison of typical breakthrough curves (blue) and isotherms (purple) for rigid microporous adsorbents (top) and “gating”‐type flexible MOFs (bottom). The gate opening and gate closing pressures are marked by A and B, respectively. The breakthrough curves for gating adsorbents have a “stepped” feature not seen in rigid adsorbents, defined by a plateau in the effluent fraction (represented by a “step height”) before the ultimate breakthrough. Reproduced with permission.^[^ ^77a^ ^]^ Copyright 2017, American Chemical Society.

To use flexible MOFs for separation, the set partial pressure of the stimulus must exceed gate opening pressure to induce the phase transition and enable the separation. Thus, in breakthrough applications, no pure gases can be obtained because the target gas exhausts until the gate opening pressure is reached. This phenomenon was named the “slipping‐off” effect.^[^ ^129^ ^]^ A comparison of a typical breakthrough curve profile of a rigid adsorbent and a representative “stepped” breakthrough curve of a “gating”‐type flexible MOF with “slipping‐off” is shown in **Figure** **40**.

**Figure 41:**
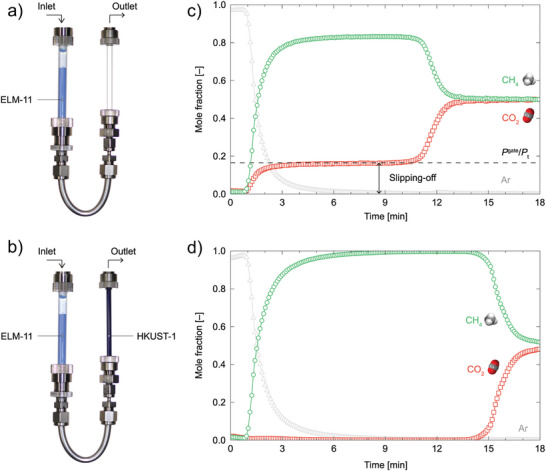
a) Pictures of the column system used by Tanaka and co‐workers in order to show the slipping‐off effect and its solution b) by combining a column of ELM‐11 and HKUST‐1. c,d) Breakthrough curves of CO_2_ and CH_4_ for the system depicted in (a) and (b), respectively. Reproduced with permission.^[^ ^22^ ^]^ Copyright 2020, The Authors, under CC BY 4.0.

Such an effect was reported several times for the breakthrough curves of flexible materials. For example, the breakthrough curves collected for CH_4_/CO_2_ on MIL‐53(Cr) exhibit distinct changes in the slope of effluent CO_2_ concentration with time.^[^ ^81^ ^]^ An even more pronounced slipping‐off breakthrough curve was observed for the separation of xylenes on MIL‐53(Al)^[^ ^33a^ ^]^ and separation of CH_4_ and CO_2_ on CID‐5, where the partial pressure of CO_2_ dropped below the gate opening pressure of the adsorbents, while it was passing the column.^[^ ^130^ ^]^

Breakthrough measurements performed on ELM‐11 also uncovered such a breakthrough step, which was discussed by Sotomayor and Lastoskie in 2017 and has also been later on found in [Co(bdp)]*_n_*.^[^ ^69^, ^77^ ^]^

Since this effect is very relevant for practical applications, more than one solution has been proposed.

i) One possible solution is to overpressure the feed gas so that the partial pressure of the opening gas remains above the gate pressure along the entire length of the column. This approach will, however, incur an energy penalty in the work input required to pressurize the feed gas.^[^ ^77a^ ^]^
ii) Another way is to directly influence the adsorption conditions by increasing or decreasing the temperature to shift the gate opening pressure. Further functionalization of the linker shows an impact if, afterward, one of the phases will be further stabilized with this functionality.^[^ ^69^ ^]^
iii) An advanced approach was demonstrated by Kitagawa and co‐workers,^[^ ^130^ ^]^ who used a solid solution of two MOFs, CID‐5 and CID‐6, to cleanly separate methane from carbon dioxide and ethane. CID‐5 has more structural flexibility than CID‐6 and exhibits selective gated adsorption for CO_2_ and C_2_H_6_ over CH_4_, whereas CID‐6 has permanent microporosity and nonselectively adsorbs all three gases. In breakthrough experiments with a 60:40 vol% CH_4_/CO_2_ mixture and a 90:10 vol% CH_4_/C_2_H_6_ mixture on CID‐5, the CH_4_ fraction in the effluent was with ≈90% well below the target due to the “slipping‐off”. Pure methane could also not be obtained for the same gas mixtures and CID‐6, because of coadsorption. However, a solid solution of CID‐5 and CID‐6 with gated adsorption characteristics for both CO_2_ and C_2_H_6_, prepared by substituting 10% of the nitroisophthalate ligands of CID‐5 with the methoxyisophthalate ligands of CID‐6, cleanly separated methane from both carbon dioxide and ethane for retention times of 8 and 25 min, respectively, in breakthrough column experiments. Hence, by tuning the gate opening pressures via framework composition, it was possible to optimize the gas separation performance under dynamic conditions.^[^ ^77^, ^130^ ^]^
iv) In 2020, a novel approach was demonstrated by Tanaka and co‐workers by successively operating columns containing flexible and rigid MOF as a guard bed (**Figure** 41).^[^ ^129^ ^]^ Significant improvement of selectivity for CO_2_ was achieved when connecting a column containing flexible ELM‐11 to a column loaded with rigid microporous HKUST‐1. In this setup, almost the entire CO_2_ could be adsorbed. The slipping‐off CO_2_ was collected by the HKUST‐1 column and thereby allowed for very high purity of the exhaust gas while maintaining a high capacity for CO_2._
v) The MOFs showing smooth, quasi‐second‐order phase transitions can also be beneficial in overcoming the slipping‐off problem.^[^ ^18b^ ^]^

## 5 Beyond Improving Working Capacity and Selectivity: Intelligent Materials and Use of Switchability

### 5.1 Thermal Management

A very important problem is the change in the temperature of the adsorption bed, leading to a reduction of adsorption capacity upon adsorption and an increase of adsorption capacity upon desorption or even preventing the complete removal of guest molecules.^[^ ^131^ ^]^

Engineering solutions to this problem can be found in adsorber design, which allows for better heat transfer. The trade‐off, however, remains between achieving high selectivity and capacity while managing the inherent challenges associated with these properties.^[^ ^129^ ^]^ Developing novel materials capable of satisfying both requirements is a key focus of ongoing research.

**Figure 42:**
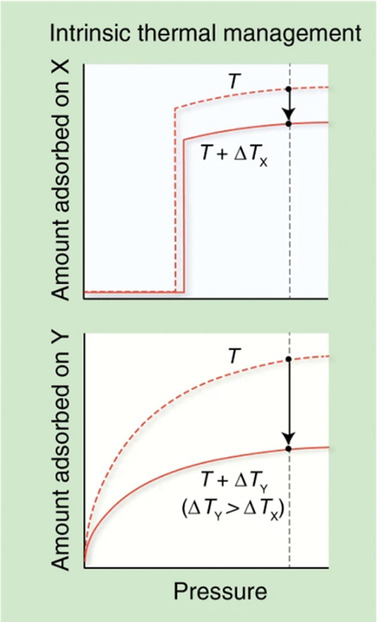
When adiabatic gas adsorption is considered, the temperature rise of the system for the flexible MOF, Δ*T_X_*, is smaller than that for the conventional adsorbent, Δ*T_Y_*, and the resulting decrease in the adsorption amount can be suppressed because of the smaller net heat of adsorption of the flexible MOF owing to its intrinsic thermal management capability. Reproduced with permission.^[^ ^22^ ^]^ Copyright 2020, The Authors, under CC BY 4.0.

One potential solution was proposed to use phase change materials (PCMs) as latent heat storing additives. PCMs are able to absorb and store some of the heat released during adsorption through a phase transition.^[^ ^131^ ^]^ The flexible MOF itself is, in a certain sense, a PCM (**Figure** **42**).^[^ ^132^ ^]^ This leads to an intrinsic thermal management upon physisorption that can be monitored in calorimetric measurements.^[^ ^26^ ^]^ The intrinsic thermal management capability of flexible MOFs should be useful for storage applications as well as for developing highly efficient PSA systems.^[^ ^118^ ^]^ Typical gas physisorption enthalpies are between −5 and −60 kJ per mol of gas,^[^ ^61^ ^]^ depending on adsorptive and surface chemistry, as well as the pore sizes of the adsorbent. The structural transition enthalpies in MOFs are connected with the thermal effects ranging between 6 and 31 kJ per mol of MOF.^[^ ^37^ ^]^

**Figure 43:**
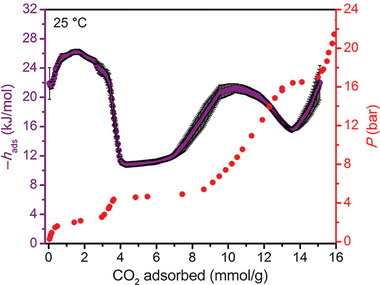
Differential enthalpies of CO_2_ adsorption in [Co(bdp)]*_n_* are shown in purple (standard errors are shown as black bars) as a function of CO_2_ loading. Local minima in adsorption enthalpy correspond to regions in which [Co(bdp)]*_n_* undergoes an endothermic structural expansion, which offsets some of the heat released upon CO_2_ adsorption and provides intrinsic thermal management. The single‐component CO_2_ adsorption isotherm (red circles) is provided for comparison. Reproduced with permission.^[^ ^69^ ^]^ Copyright 2018, American Chemical Society.

In [Co(bdp)]*_n_*_,_ differential enthalpies of CH_4_ and CO_2_ adsorption reveals significant reductions in heat released upon adsorption during the discrete, endothermic structural phase changes relative to the regions between these phase changes (**Figure** **43**). For comparison, the isostructural rigid MOFs [Ni(bdp)]*_n_* and [Zn(bdp)]*_n_*, display differential enthalpies of CO_2_ adsorption of −20 kJ mol^−1^ at zero coverage.^[^ ^133^ ^]^ Notably, these values are very close to those observed for [Co(bdp)]*_n_* at the pressures between the CO_2_‐induced phase transition. During the first phase transition, [Co(bdp)]*_n_* shows values in the range of −24 to −26 kJ mol^−1^, which are significantly lower in magnitude than what might be expected. During the second and third CO_2_‐induced phase transitions, the magnitude of adsorption enthalpy plummets dramatically, reaching values as small as −11 and −16 kJ mol^−1^, respectively (Figure 43). Thus, the structural phase changes of [Co(bdp)]*_n_*, because of their endothermic nature, can substantially mitigate the amount of heat that must be dissipated during adsorption.

For ELM‐11, the molar integral heats of adsorption in the phases with increasing porosity are estimated by GCMC simulations to be 40.8, 38.3, and 33.6 kJ per mol CO_2_, respectively. The transition enthalpies were determined to be 21.9 kJ per mol CO_2_ for the gate closing at higher pressures and 25.5 kJ per mol CO_2_ for the gate closing at lower pressure, which suggests that both closings are endothermic processes and that more heat per mole of CO_2_ is removed from the system for the gate closing at lower pressure than for that at the higher pressures.

Hiraide et al.,^[^ ^129^ ^]^ in their study on ELM‐11, demonstrated that during the adsorption of an equimolar CO_2_/CH_4_ gas mixture at 500 kPa and 298 K, the host material undergoes an endothermic expansion with an associated enthalpy change of 55.7 J g^−1^. Concurrently, the exothermic enthalpy change due to the adsorption of the gas mixture is 135.3 J g^−1^. The net heat effect is decreased to 79.6 J g^−1^, indicating that 41% of the exothermic heat is offset by the endothermic process.

Thus, the structural transitions in flexible adsorbents offer obvious advantages for thermal management compared to rigid adsorbents.

## 6 Conclusion

Flexible MOFs are emerging materials for advanced applications in energy storage and gas separation thanks to their unique adsorption properties and responsive behavior. The flexibility of the frameworks essentially contributes to achieving exceptionally high selectivity in separation processes and improves deliverable storage capacity. These materials offer great potential for tackling challenging (isotope) separations that would be difficult or even impossible utilizing rigid structures. The responsivity of MOFs can be fine‐tuned by adjusting the metal type, linker substituents, or by employing mixed metal or mixed linker strategies.

When designing separation processes, it is crucial to consider not only the single gas adsorption isotherms of both components involved but also the desorption branches (as discussed in Section 4), as this is essential for accurately predicting or leveraging the enhanced separation capabilities of flexible MOFs. Additionally, the temperature‐dependent behavior of these flexible frameworks must be carefully considered, as it plays a key role in their performance under different conditions.

For column‐based applications, MOFs with minimal volume changes are generally preferable to ensure stable operation, but it has been shown, that also flexible MOFs can be shaped while maintaining their storage capacity and flexibility, allowing for greater adaptability in industrial applications. Additionally, their intrinsic thermal management capabilities further enhance their suitability for demanding energy storage and gas separation processes. With their versatile and tunable properties, flexible MOFs are poised to revolutionize these two fields in the future.

## Conflict of Interest

The authors declare no conflict of interest.
